# Comparative efficacy of exercise interventions on depressive symptoms and related outcomes in patients with Alzheimer’s disease, cognitive impairment, and Parkinson’s disease: a systematic review and network meta-analysis

**DOI:** 10.3389/fphys.2026.1825740

**Published:** 2026-05-25

**Authors:** Zixin Chen, YuanXi Zhang, Xiaojie Zhu, Huihong Wu, Tongwu Yu, Ziwang Liu

**Affiliations:** 1 Department of Encephalopathy, Dongzhimen Hospital Affiliated to Beijing University of Chinese Medicine, Beijing, China; 2 Beijing University of Chinese Medicine, Beijing, China; 3 Capital University of Physical Education and Sports, Beijing, China

**Keywords:** Alzheimer’s disease, cognitive impairment, depression, exercise interventions, network meta-analysis, Parkinson’s disease

## Abstract

**Background:**

Patients with Alzheimer’s disease, cognitive impairment, and Parkinson’s disease often experience depressive symptoms, which negatively impact quality of life and disease management. Exercise is an important non-pharmacological treatment in these populations; however, the relative efficacy of different exercise modalities remains unclear. This study used a systematic review and network meta-analysis to compare the effects of various exercise interventions on depressive symptoms and related outcomes.

**Methods:**

We systematically searched Chinese and English databases for randomized controlled trials evaluating exercise interventions in patients with Alzheimer’s disease, cognitive impairment (including mild cognitive impairment and dementia), and Parkinson’s disease. The primary outcome was depressive symptoms, assessed by using Geriatric Depression Scale (GDS) and Beck Depression Inventory (BDI); the secondary outcomes were cognitive function and motor function, assessed by using Mini-Mental State Examination (MMSE) and Unified Parkinson’s Disease Rating Scale Part III (UPDRS III), respectively. Network meta-analysis was performed using Stata 16.0, and supplementary pairwise meta-analyses by disease type were conducted for the primary outcomes. RevMan 5.4 was used to assess the risk of bias, and the CINeMA framework was used to evaluate the credibility of the evidence.

**Results:**

A total of 23 randomized controlled trials involving 1,596 subjects were included. For the primary outcomes, no exercise modality showed an overall statistically significant advantage over the control group in improving depressive symptoms. For the secondary outcome, cognitive exercise significantly improved the MMSE scores compared with daily activities (MD = 3.50, 95% CI [2.29, 4.71]). For UPDRS III, no significant overall difference was observed between exercise interventions and the control group.

**Conclusion:**

The available evidence does not provide stable or modestly consistent support for a clear overall benefit of different exercise interventions on depressive symptoms in patients with Alzheimer’s disease, cognitive impairment, and Parkinson’s disease. Cognitive exercise may help improve cognitive function, whereas the potential benefits of multicomponent exercise for motor function in Parkinson’s disease should be interpreted with caution. In the future, large randomized controlled trials with clearer stratification, specific protocols, and standardized exercise prescriptions are needed to further define the optimal populations and intervention effects of different exercise modalities.

**Systematic review registration:**

https://www.crd.york.ac.uk/prospero/, identifier CRD420261280926.

## Introduction

The prevalence of depression among patients with Alzheimer’s disease is approximately 38%, and the two conditions may share common risk factors and a bidirectional relationship (Huang et al., 2024). Neuroinflammation, metabolic alterations, and neurodegenerative processes in Alzheimer’s disease may disrupt serotonergic projections to the forebrain and noradrenergic projections to the neocortex and hippocampus, thereby promoting the development of depression in this population (Haussmann and Donix, 2023). Depressive symptoms are also common in individuals with cognitive impairment, with an overall pooled prevalence of approximately 32% (Ismail et al., 2017). Cognitive impairment has been linked to the activation of the NLRP3 inflammasome, which further activates downstream pro-inflammatory cytokines such as interleukin-1β (IL-1β), thereby triggering chronic inflammatory responses that may contribute to the pathophysiology of depression (Li et al., 2023). In Parkinson’s disease, depression is a common non-motor symptom and is usually associated with alterations in catecholamines, serotonin, and dopamine (Allain et al., 2000). Moreover, greater depression severity may aggravate neurological impairment, reduce quality of life, increase mortality risk, and increase caregiver burden (Stogmann et al., 2016; Orgeta et al., 2022).

Current treatments for depression include psychotherapy, antidepressant therapy, or a combination of both; however, psychotherapy is costly, and antidepressant therapy may cause side effects and is associated with relapse and withdrawal symptoms after discontinuation. Physical activity, as a promising non-pharmacological treatment, is playing an increasingly important role in improving depressive symptoms and preventing cognitive decline, and many randomized controlled trials have reported a positive effect of exercise on depressive symptoms (Mollinedo Cardalda et al., 2019; Morres et al., 2019; Heissel et al., 2023). Biologically, exercise is beneficial for metabolic processes, activating adaptive mechanisms based on the regulation of tissue plasticity processes, promoting the circulation of neurotrophic factors and improving cerebral blood flow, as well as reducing oxidative stress and inflammatory factors, modulating the neuroendocrine system and improving depressive symptoms (Kandola et al., 2019; Amato et al., 2024).

It is important to note that different exercise modalities may influence depressive symptoms and related outcomes through different mechanisms. Aerobic exercise may increase the levels of neuroprotective factors in the brain, such as brain-derived neurotrophic factor (BDNF) and glial-cell-line-derived neurotrophic factor (GDNF). Resistance training may improve blood flow and cerebral oxygenation, thereby promoting the synthesis and release of BDNF (Setayesh and Mohammad Rahimi, 2023; Wilson et al., 2025). Mind–body exercise, such as yoga, may act through the hypothalamic–pituitary–adrenal (HPA) axis, gamma-aminobutyric acid (GABA)-related pathways, autonomic regulation, limbic system activity, and inflammatory and endocrine responses, thereby helping to alleviate depressive symptoms (R et al., 2023). Cognitive exercise may promote neurogenesis and stimulate growth-related biomarkers, thereby positively influencing both physical and cognitive function in the peripheral and central nervous systems (e.g., insulin-like growth factor-1 [IGF-1]) (Falck et al., 2019). Through the integration of aerobic, resistance, balance, and coordination training, multicomponent exercise may simultaneously influence multiple pathways, including neural plasticity, cerebral blood flow, energy metabolism, inflammatory responses, and neuroendocrine regulation, thereby contributing to improvements in overall function and depressive symptoms.

However, despite the potential benefits of exercise interventions for depressive symptoms, the available evidence focuses mainly on answering the question of whether exercise is effective or not, and there is still a lack of systematic evidence comparing multiple exercise modalities. Traditional paired meta-analyses usually only integrate limited direct comparative evidence, making it difficult to simultaneously compare the relative efficacy of multiple interventions and to provide a more targeted basis for exercise prescription in clinical practice. In contrast, network meta-analyses are a better way to accurately quantify differences in interventions because they simulate both direct and indirect comparisons between interventions and are better suited to assessing the relative efficacy of different exercise modalities on depressive symptoms and related outcomes.

Previous systematic reviews and meta-analyses have explored the effects of exercise interventions on depression and cognitive function, but most have focused on single-disease populations—for example, some studies suggest that exercise prescriptions may help guide the treatment of depression (Noetel et al., 2024), whereas studies in patients with Alzheimer’s disease support the view that exercise may improve cognitive decline and reduce neuropsychiatric symptoms (López-Ortiz et al., 2021). Meta-analyses have also shown that physical activity may alleviate depressive symptoms in patients with Parkinson’s disease (Kim et al., 2023). However, important gaps remain in the current evidence. First, most studies on depression have been conducted in the general depressed population, and their findings may not be directly generalizable to patients with Alzheimer’s disease, cognitive impairment, or Parkinson’s disease. Second, studies in Alzheimer’s disease and cognitive impairment have mainly focused on cognitive outcomes and the overall effects of exercise, with relatively few studies directly comparing different exercise modalities using depressive symptoms as the primary endpoint. Third, because Alzheimer’s disease, cognitive impairment, and Parkinson’s disease are common clinical conditions with a high prevalence of comorbid depression, which, in turn, affects prognosis and disease management, there is still limited evidence comparing the relative efficacy of different exercise modalities for depression within a unified analytical framework. Therefore, the key gap in the literature is not whether exercise is beneficial, but whether different exercise modalities differ in their relative efficacy in specific clinical populations when depressive symptoms are treated as the primary outcome.

Therefore, the present study included randomized controlled trials in patients with Alzheimer’s disease, cognitive impairment, and Parkinson’s disease. Using systematic review and network meta-analysis, we compared the relative efficacy of different exercise modalities with depressive symptoms as the primary outcome and further assessed their effects on cognitive and motor function and other related outcomes, with a view to providing the basis for the clinical development of a more targeted exercise prescription.

## Materials and methods

### Search strategy

We conducted a comprehensive literature search using both automated and manual methods in several databases, including PubMed, the Cochrane Library, Embase, Web of Science, CNKI, Wanfang Data, VIP Database, and the Chinese Biomedical Literature Database. The search was conducted from database inception to January 10, 2026. Literature selection and data extraction were performed independently by two reviewers (CZX and ZYX), and any discrepancies were resolved through consultation with a third reviewer (LZW). We chose databases that are commonly used in contemporary meta-analyses, covering randomized controlled trials related to the topic, in both Chinese and English. In addition, we conducted an exhaustive search of existing meta-analyses and reviews to ensure that the literature was included as comprehensively as possible. Initially, titles and abstracts of identified papers were reviewed to exclude irrelevant studies. Subsequently, the full text of the remaining entries was assessed to determine their eligibility for inclusion. The study selection process followed the guidelines of the Preferred Reporting Items for Systematic Reviews and Meta-Analyses (PRISMA) (Page et al., 2021).

### Inclusion and exclusion criteria

Our inclusion and exclusion criteria were based on the PICOS framework and are shown in **Table 1**. The inclusion criteria were as follows: (1) The type of study was a randomized controlled trial; (2) The age of the participants was ≥18 years; (3) The study participants were adults with clinically diagnosed Alzheimer’s disease, cognitive impairment, or Parkinson’s disease, of which the cognitive impairment mainly included those with mild cognitive impairment and those with dementia, and were defined according to clinical diagnostic criteria or cognitive assessment tools used in the study; (4) The study reported at least one outcome relevant to this review, including Geriatric Depression Scale (GDS), Beck Depression Inventory (BDI), Mini-Mental State Examination (MMSE), or Unified Parkinson’s Disease Rating Scale Part III (UPDRS III); (5) The intervention group received at least one type of exercise intervention, whereas the control group received usual care, daily activities, or no intervention. Exercise interventions in this review included aerobic exercise, resistance exercise, multicomponent exercise, mind–body exercise, balance stretching, and cognitive exercise.

**Table 1:** PICOS criteria.

Criteria	Explanation
Population	Adults (≥18 years) diagnosed with Alzheimer’s disease, cognitive impairment (including MCI and dementia), or Parkinson’s disease with depression
Intervention	Specific exercise (Aerobic Exercise, Strength Exercise, etc.)
Comparator	Conventional therapy, routine, or no intervention
Outcomes	GDS, BDI, MMSE, UPDRS III
Study design	RCTs

The exclusion criteria were as follows: (1) studies with duplicate publications or duplicate reports of data, (2) animal experiments, basic research, or other nonclinical studies, (3) meeting abstracts, reviews, letters, case reports, and studies for which full-text or complete outcome data were not available, and (4) non-randomized controlled trials.

### Data extraction and quality assessment

Data extraction and quality assessment were done independently by two reviewers (CZX and ZYX), and discrepancies were resolved by a third reviewer (LZW). Data were extracted and subsequently cross-validated. In the case of discrepancies, a third researcher (LZW) was asked to adjudicate. The following information was collected from each article: year of the article, name of the first author, age of the subjects, gender, study design, and values of the outcome metrics. All data were derived from the final outcome measures. Risk of bias in RCTs was assessed using Risk of Bias Assessment Tool for RCTs recommended in Cochrane Handbook, version 6.5.0 (online at https://www.cochrane.org/); certainty of evidence was assessed using the CINeMA (Confidence in Network Meta-Analysis) framework (https://cinema.ispm.unibe.ch/). The framework, based on the GRADE methodology and applicable to network meta-analysis, assesses the credibility of the evidence for each pairwise comparison in terms of within-study bias, reporting bias, indirectness, imprecision, heterogeneity, and inconsistency.

The interventions involved in the data extraction process included moderate-intensity aerobic activity, multicomponent exercise training, motor cognitive dual-task training, structured physical exercise programs, yoga, Tai Chi, running, aerobics, balance stretching, Nordic walking, cycling, cup-stacking exercises, strength training, and Qigong. To improve comparability across studies and with reference to the Physical Activity Guidelines for Americans (Piercy et al., 2018) and previous systematic reviews, exercise interventions were classified *a priori* according to their dominant training component, primary target, and mode of delivery, rather than by specific activity name, as follows: (1) aerobic exercise (AE): interventions designed to improve cardiovascular fitness, including walking, running, and cycling; (2) resistance exercise (RE): interventions designed to increase muscular strength and endurance, such as training with elastic bands and weightlifting equipment; (3) multicomponent exercise (ME): complex interventions integrating two or more core training components, such as aerobic, resistance, balance, coordination, or flexibility training. Although the specific combinations, training focus, and dosage of multicomponent exercise varied across studies, their common feature was the simultaneous integration of multiple core training components targeting several functional domains; therefore, they were classified into the same intervention category for comparison; (4) mind–body exercise (MBE): interventions involving a series of controlled movements that emphasize the interaction between mind and body, such as Tai Chi, yoga, and dance-based exercise; (5) balance stretching (BS): stretching-based interventions that incorporate balance-training elements to improve flexibility while enhancing balance and coordination; and (6) cognitive exercise (CE): movement-based interventions that incorporate explicit cognitive demands, with a primary focus on processes such as attention, executive control, and task switching. Examples include motor–cognitive dual-task training and cup-stacking exercises. Although different protocols may vary in specific task formats and primary mechanisms of action, their common feature was the introduction of cognitive load into motor training; therefore, they were classified into the same intervention category for comparison.

### Statistical analysis

All statistical analyses were performed using Stata 16.0. The network meta-analysis was implemented using the mvmeta package, and supplementary pairwise meta-analyses were conducted for the primary outcomes. For continuous variables, mean difference (MD) was used as the effect size measure, with 95% confidence intervals (CIs). Risk-of-bias assessments were organized and presented using RevMan 5.4, heat maps were generated using R 4.3.1, and certainty of evidence was evaluated using the CINeMA framework.

Considering the potential clinical and methodological differences between the included studies in terms of population characteristics, intervention programs, duration, and control measures, a random-effects model was used as the primary analytical approach for the network meta-analysis. Heterogeneity was assessed descriptively using the *I*² statistic and combined with clinical and methodological differences, in which *I*² >50% suggested the existence of large heterogeneity; when necessary, sensitivity analysis or subgroup analysis was used to further explore the potential sources, and statistical test was performed at the level of *α* = 0.05 for significance.

To further assess the possible clinical heterogeneity by disease type, the present study conducted a supplementary pairwise meta-analysis for the primary outcomes (GDS and BDI) by disease type. Considering the limited number of studies with different exercise modalities within each disease subgroup, the present analysis used the comparison of exercise interventions versus controls as the basic framework, and the random-effects model was also used for the combined analyses. For multi-arm studies, to avoid double-counting of shared controls, only one exercise intervention–control comparison that met the analytic purpose was retained for inclusion in the supplementary pairwise meta-analysis.

The plausibility of the transitivity assumption was assessed by comparing potential effect modifiers, including disease type, age, baseline depression severity, intervention duration, training frequency or dose, and type of control group, before network construction. The relevant study characteristics and interventions are summarized in **Supplementary Tables 1**, **S2**. Local inconsistency in the network was tested by node-splitting; consistency within closed loops was assessed by the inconsistency factor (IF), and direct comparisons were considered to have no significant inconsistency with indirect comparisons when the 95% CI of the IF contained 0. There was no significant inconsistency between direct and indirect comparisons. The relative efficacy of each intervention was demonstrated by league table, and the interventions were ranked using the surface under the cumulative ranking curve (SUCRA), with SUCRA values ranging from 0% to 100%. The higher the value, the higher the probability that the intervention is the best program.

For multi-arm randomized controlled trials, this study retained their original multi-arm structure in the network meta-analysis and modeled them using statistical methods that took into account the correlation induced by the shared control group to avoid overestimation of precision and estimation bias due to repeated counting of the same control group. For studies containing two or more intervention arms that met the inclusion criteria, the mean, standard deviation, and sample size of each intervention arm were included in the network model with the original arm-level data.

## Results

### Literature search details

**Figure 1:**
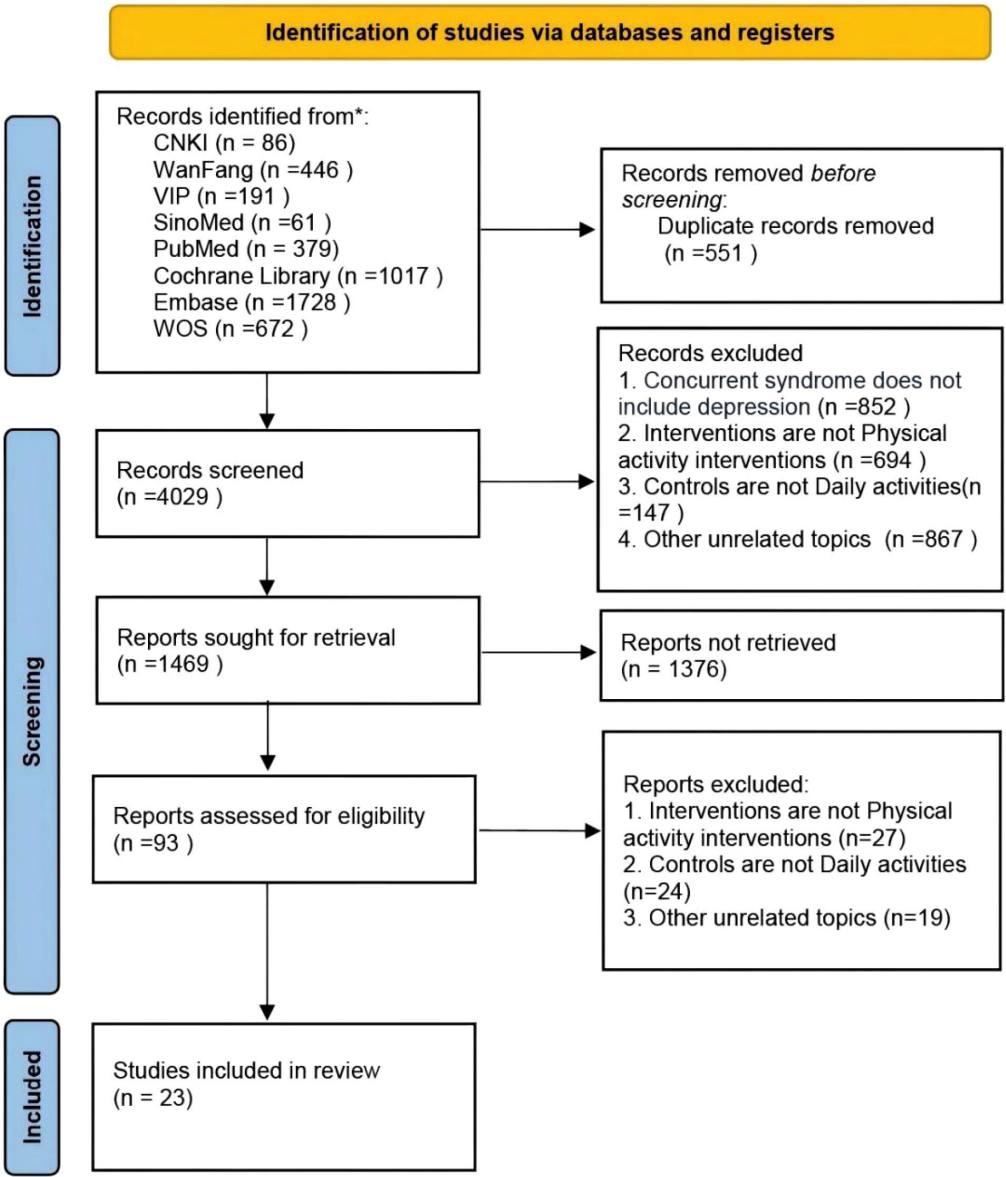
Document screening process and results.

**Figure 1** illustrates the literature screening process. Initially, a total of 4,580 studies were retrieved from the database. Duplicate articles and studies that did not meet the inclusion criteria were subsequently excluded. After a thorough review of the texts, 23 articles were found to meet the inclusion criteria and were included in the meta-analysis. Five articles were in Chinese and 18 in English, with a total of 1,596 participants. The included studies were published from database inception to January 2026. For a detailed description of the search terms used, please refer to Appendix S1 of the **Supplementary Material**.

### Classification of the study characteristics

We included a total of 23 studies (Li et al., 2012; Cheon et al., 2013; Cugusi et al., 2015; Altmann et al., 2016; Bega and Stein, 2016; Hoffmann et al., 2016; Abd El-Kader and Al-Jiffri, 2017; Liu et al., 2017; Cheung et al., 2018; Henskens et al., 2018; Lee et al., 2018; Harper et al., 2019; Huang et al., 2019; Solla et al., 2019; Xie et al., 2019; Liu et al., 2020; Wang et al., 2020; Zhu et al., 2020; Wu et al., 2021; Menengiç et al., 2022; Bardopoulou et al., 2023; Jiang et al., 2023; Zhou et al., 2024). All studies were randomized controlled trials comparing conventional therapy with one of six exercise interventions: aerobic exercise, resistance exercise, multicomponent exercise, mind–body exercise, balance stretching, or cognitive exercise.

To assess the plausibility of the transitivity assumption, a structured comparison of the type of illness, baseline depression severity, duration of intervention, frequency or dose of training, and type of control group of the included studies was performed, and the results are shown in **Supplementary Tables 1**, **S2**. Overall, no significant systematic imbalance was observed, but a certain amount of clinical heterogeneity still existed in some of the studies, and the results should be interpreted with caution.

### Literature quality evaluation

**Figure 2:**
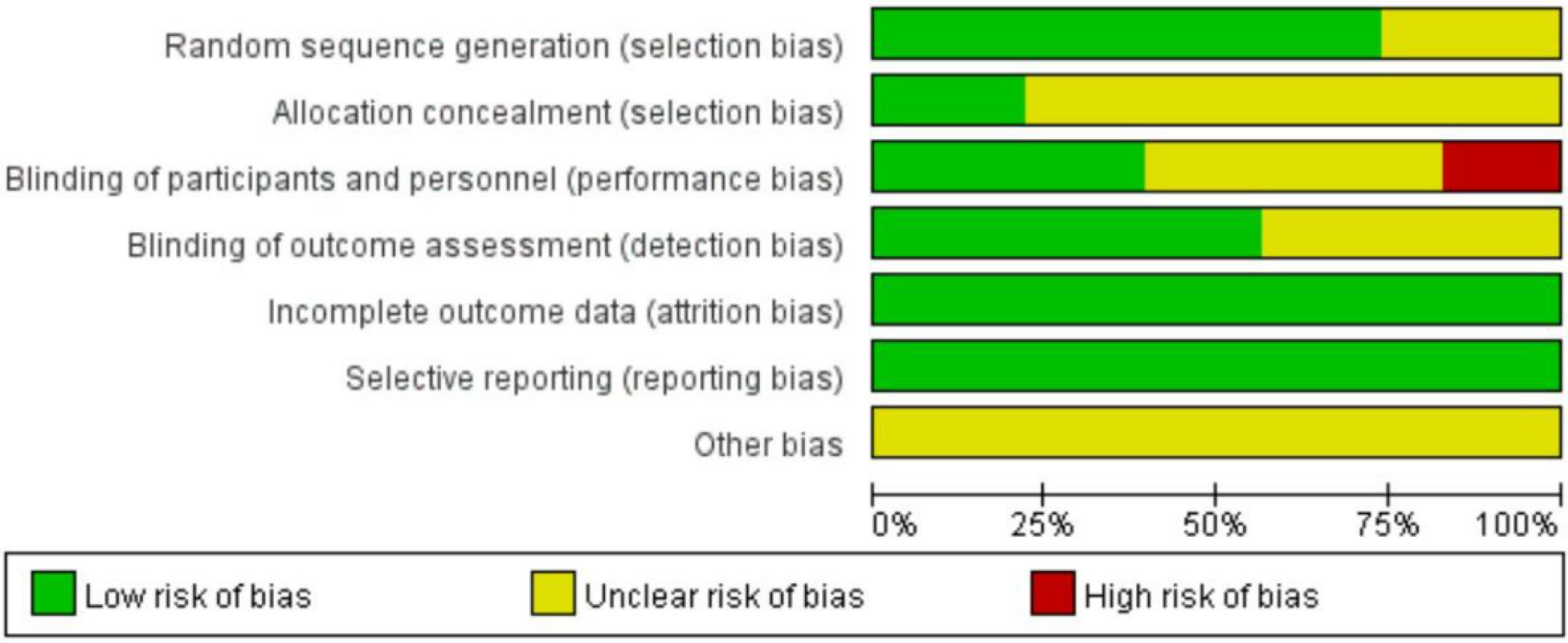
Risk of bias assessment included in the study.

We assessed the methodological quality of the 23 randomized controlled trials included in the meta-analysis. Two authors (CZX and ZYX) independently evaluated the risk of bias, and disagreements were resolved by a third author (LZW). Regarding sequence generation, 17 studies were judged as low risk because they used random number tables or computer-generated randomization methods (Li et al., 2012; Cugusi et al., 2015; Bega and Stein, 2016; Hoffmann et al., 2016; Abd El-Kader and Al-Jiffri, 2017; Cheung et al., 2018; Henskens et al., 2018; Lee et al., 2018; Huang et al., 2019; Solla et al., 2019; Liu et al., 2020; Wang et al., 2020; Zhu et al., 2020; Wu et al., 2021; Menengiç et al., 2022; Jiang et al., 2023; Zhou et al., 2024), whereas six studies mentioned randomization without providing sufficient details and were therefore rated as unclear (Cheon et al., 2013; Altmann et al., 2016; Liu et al., 2017; Harper et al., 2019; Xie et al., 2019; Bardopoulou et al., 2023). For allocation concealment, five studies reported adequate methods and were rated as low risk (Cheung et al., 2018; Huang et al., 2019; Wang et al., 2020; Wu et al., 2021; Bardopoulou et al., 2023), while the remaining 18 studies did not provide sufficient information and were judged as unclear (Li et al., 2012; Cheon et al., 2013; Cugusi et al., 2015; Altmann et al., 2016; Bega and Stein, 2016; Hoffmann et al., 2016; Abd El-Kader and Al-Jiffri, 2017; Liu et al., 2017; Henskens et al., 2018; Lee et al., 2018; Harper et al., 2019; Solla et al., 2019; Xie et al., 2019; Liu et al., 2020; Zhu et al., 2020; Menengiç et al., 2022; Jiang et al., 2023; Zhou et al., 2024). Because of the nature of exercise interventions, participant blinding was not feasible in four studies and was therefore assessed as high risk (Li et al., 2012; Cheon et al., 2013; Abd El-Kader and Al-Jiffri, 2017; Wu et al., 2021). A total of 13 studies explicitly reported blinding of outcome assessors and were rated as low risk for detection bias (Altmann et al., 2016; Bega and Stein, 2016; Cheung et al., 2018; Henskens et al., 2018; Lee et al., 2018; Solla et al., 2019; Liu et al., 2020; Wang et al., 2020; Zhu et al., 2020; Wu et al., 2021; Bardopoulou et al., 2023; Jiang et al., 2023; Zhou et al., 2024), whereas the remaining 10 studies did not report this information and were judged as unclear (Li et al., 2012; Cheon et al., 2013; Cugusi et al., 2015; Hoffmann et al., 2016; Abd El-Kader and Al-Jiffri, 2017; Liu et al., 2017; Harper et al., 2019; Huang et al., 2019; Xie et al., 2019; Menengiç et al., 2022). All studies provided complete outcome data. Selective reporting was judged as low risk in all studies because the Methods and Results sections were consistent and outcomes were adequately reported. However, other potential sources of bias remained unclear. Risk-of-bias assessment was performed using RevMan 5.4, and the results are shown in **Figure 2**. Details of the risk-of-bias assessments for each study are presented in **Supplementary Figure 1**.

### Network meta-analysis

#### Primary outcomes

##### Depressive symptoms

**Figure 3:**
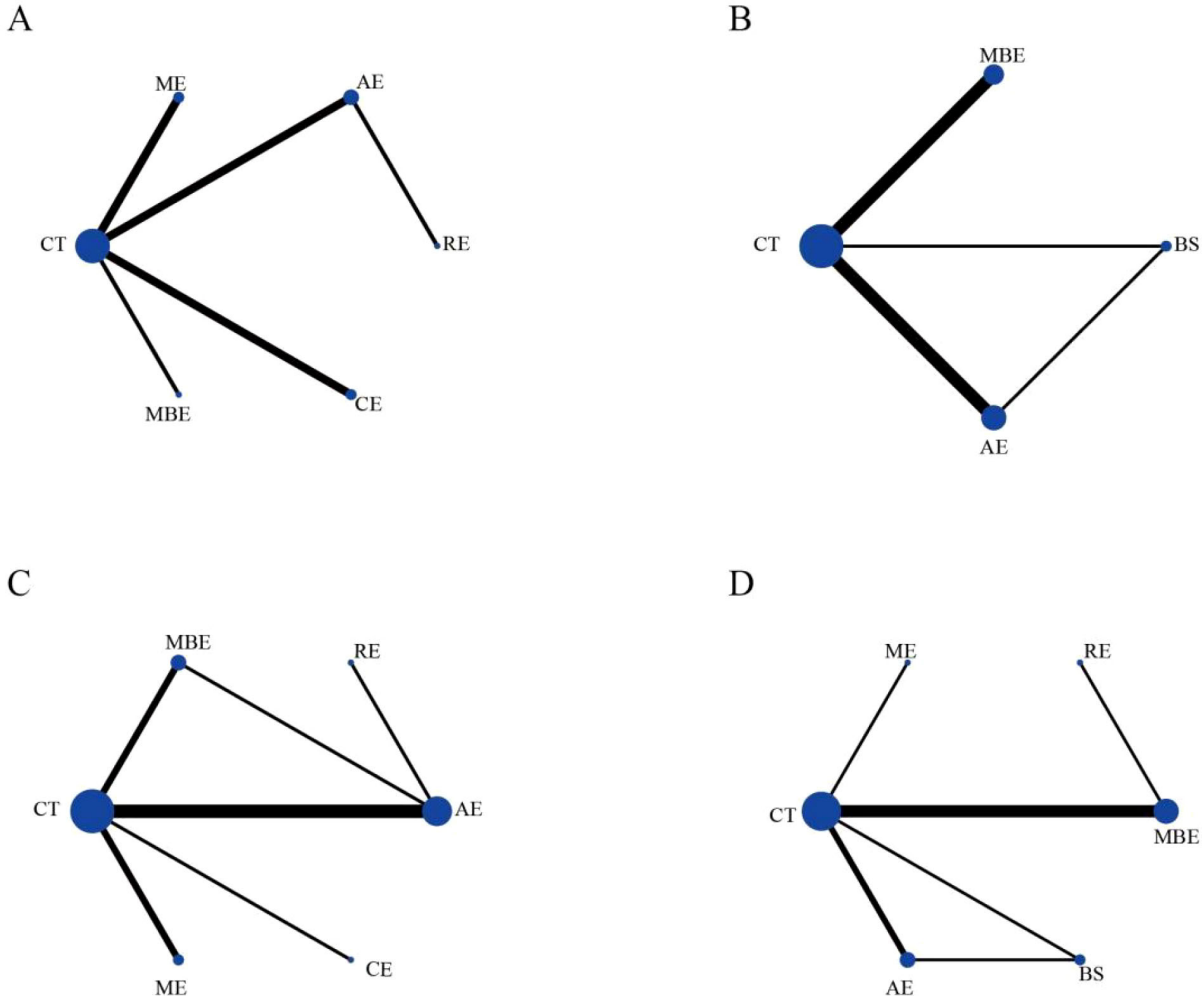
Evidence network of all papers on different exercises. **(A)** GDS, **(B)** BDI, **(C)** MMSE, **(D)** UPDRS III. The thickness of the lines represents the number of studies, and the sizes of the nodes indicate the total sample sizes for each exercise. GDS, Geriatric Depression Scale; BDI, Beck Depression Inventory; MMSE, Mini-Mental State Examination; UPDRS III, Unified Parkinson’s Disease Rating Scale III.

**Figure 4:**
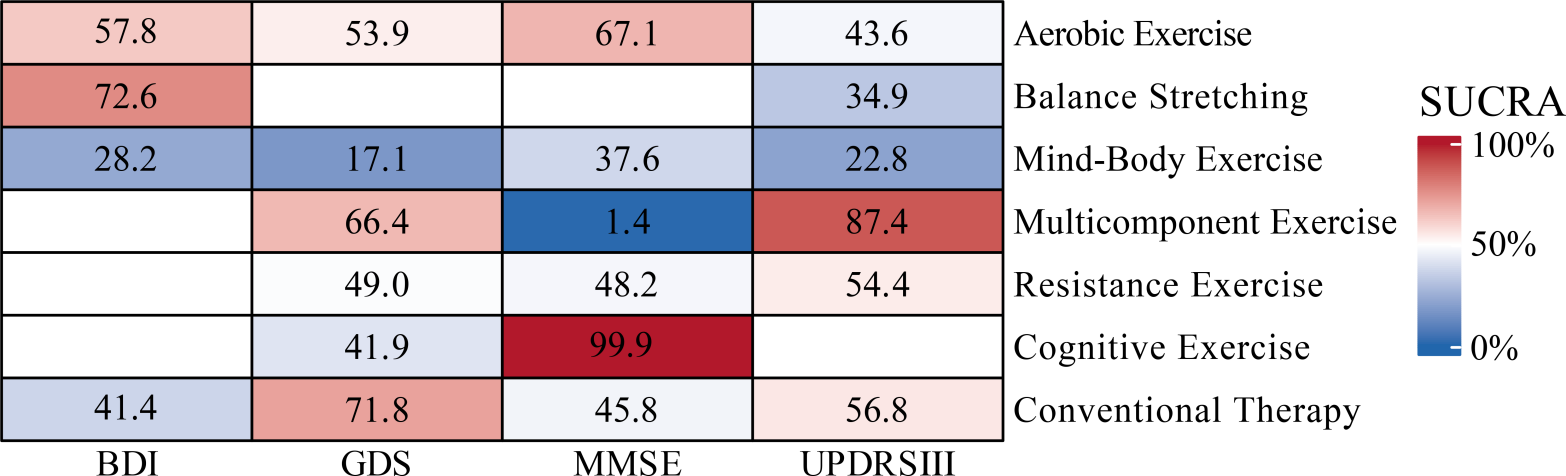
Heat map of SUCRA values for various exercise treatments. The SUCRA values, ranging from 0 to 100%, indicate the likelihood of each exercise being the most effective treatment. BDI, Beck Depression Inventory; GDS, Geriatric Depression Scale; MMSE, Mini-Mental State; UPDRS III, Unified Parkinson’s Disease Rating Scale III.

**Figure 5:**
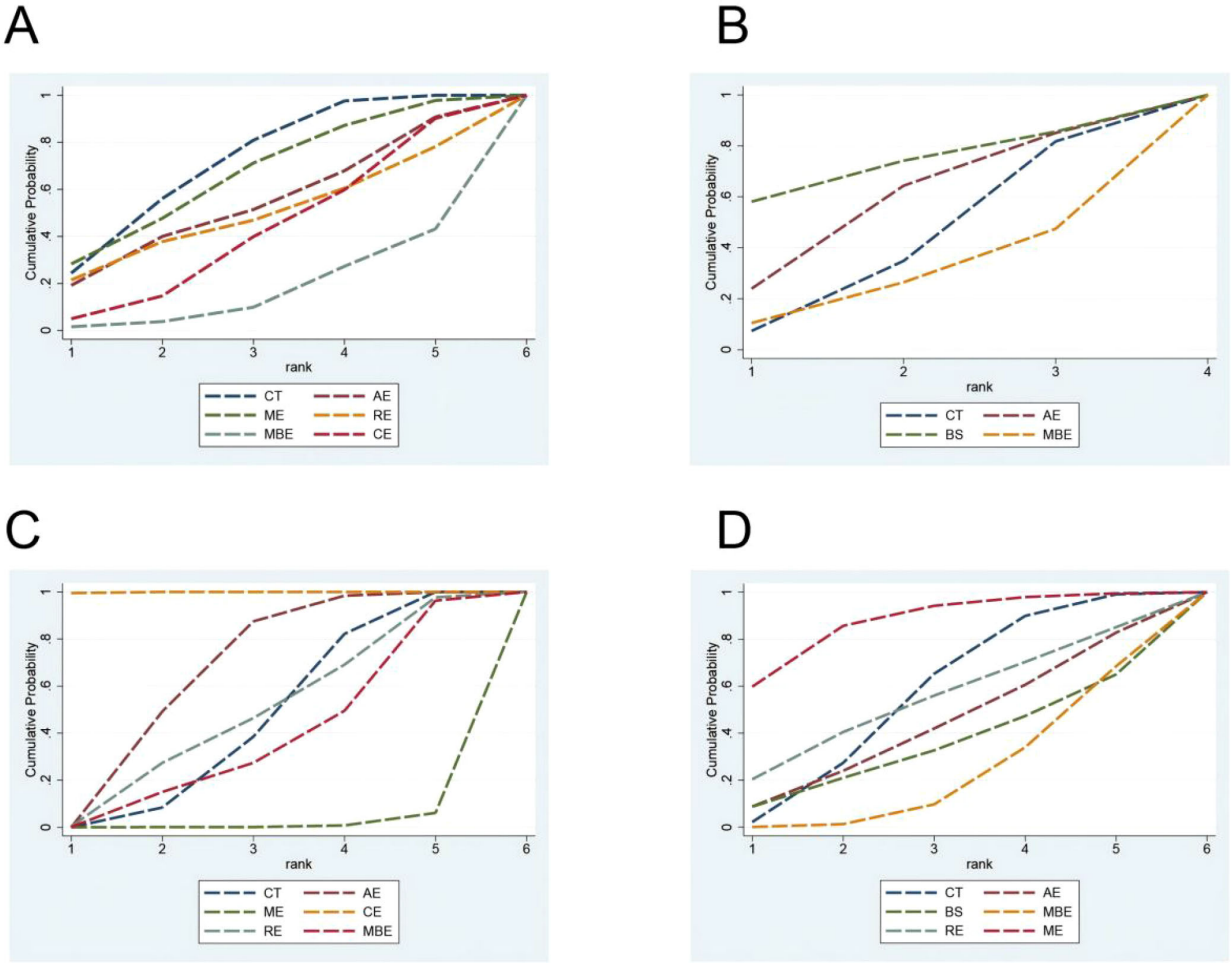
SUCRA values for principal outcomes. **(A)** GDS, **(B)** BDI, **(C)** MMSE, **(D)** UPDRS III. GDS, Geriatric Depression Scale; BDI, Beck Depression Inventory; MMSE, Mini-Mental State Examination; UPDRS III, Unified Parkinson’s Disease Rating Scale III.

Geriatric Depression Scale (GDS): A total of eight studies documented the Geriatric Depression Scale scores of patients who underwent exercise interventions, covering 460 participants and six physical activity intervention modalities, and the network relationships between these interventions are shown in **Figure 3A**. The consistency model was considered appropriate for the NMA if the inconsistency model was tested at *P >*0.05. The NMA showed that, with respect to the Geriatric Depression Scale, no statistically significant differences were observed in the two-way comparisons of the six physical activity intervention modalities compared with conventional therapy (CT) (*P* > 0.05). Details of the results of the two-way comparisons are shown in **Supplementary Table 3**. According to the SUCRA analysis, there were some differences in the rankings of the intervention modalities in terms of the GDS outcomes, among which CT had the highest SUCRA value (71.8%), followed by multicomponent exercise (66.4%). However, since no statistically significant differences were observed in the network comparisons, the ranking results should be considered exploratory only and should not be used to infer clear clinical superiority of any intervention. The SUCRA values for the other therapies are shown in **Figures 4**, **5A**.

Beck Depression Inventory (BDI): A total of eight studies recorded scores on the Beck Depression Inventory in patients undergoing exercise interventions, covering 222 participants and four types of exercise interventions, and the network relationships between these interventions are shown in **Figure 3B**.

Consistency modeling was used by NMA. The analysis showed that, regarding the BDI, the four exercise interventions were compared pairwise with CT, and although the exercise interventions showed a tendency to reduce Beck Depression Inventory scale scores, this difference did not reach statistical significance (*P* > 0.05). Details of the results of the pairwise comparisons are presented in **Supplementary Table 4**. Closed loops were formed among the interventions, and loop inconsistency was assessed. The combination of CT, AE, and BS formed a closed loop (IF = 1.283, 95% CI [0, 7.49], *P* = 0.685), suggesting no statistically significant inconsistency between direct and indirect evidence. Therefore, the consistency model was considered appropriate for subsequent estimation. According to the SUCRA results, BS (72.6%) and AE (57.8%) ranked relatively high in the BDI outcomes, but since no statistically significant differences were observed in the network comparison, the relevant ranking results can only be used as an exploratory reference. The SUCRA values for other therapies are shown in **Figures 4**, **5B**.

#### Secondary outcomes

##### Cognitive function

Mini-Mental State Examination (MMSE): A total of nine studies recorded scores on the Mini-Mental State Examination in patients undergoing exercise interventions, covering 655 participants and six physical activity intervention modalities, and the network relationships between these interventions are shown in **Figure 3C**.

Using a consistency model, the NMA showed that cognitive exercise was significantly associated with better MMSE scores than several other interventions. Specifically, cognitive exercise was associated with significantly higher MMSE scores than CT (MD = 3.50, 95% CI [2.17, 4.83]), resistance exercise (MD = 3.48, 95% CI [0.98, 5.99]), aerobic exercise (MD = 2.98, 95% CI [1.35, 4.62]), multicomponent exercise (MD = 6.30, 95% CI [3.94, 8.66]), and mind–body exercise (MD = 3.89, 95% CI [1.55, 6.24]) (all *P* < 0.05). In pairwise comparisons among the remaining interventions, aerobic exercise also showed a statistically significant advantage over multicomponent exercise. A closed loop was formed among CT, AE, and MBE, and loop inconsistency testing showed no statistically significant inconsistency (IF = 1.378, 95% CI [0, 4.91], *P* = 0.444). Therefore, the consistency model was considered appropriate. Details of the pairwise comparison results are shown in **Supplementary Table 5**. According to the SUCRA analysis, cognitive exercise ranked highest for MMSE (SUCRA = 99.9%), followed by aerobic exercise (SUCRA = 67.1%); the SUCRA values for the remaining interventions are shown in **Figures 4**, **5C**.

##### Motor function

UPDRS III: A total of eight studies documented UPDRS III scores in patients who underwent exercise interventions, covering 259 participants and six physical activity intervention modalities, and the network relationships between these interventions are shown in **Figure 3D**. A consistency model was considered appropriate for the NMA because the inconsistency test did not indicate significant inconsistency (*P* > 0.05). The NMA showed that none of the six exercise interventions was significantly superior to the control group overall for UPDRS III. However, multicomponent exercise showed a statistically significant advantage over mind–body exercise (MD = -5.89, 95% CI [-11.67, -0.11], *P* < 0.05). No statistically significant differences were observed in the other pairwise comparisons. A closed loop was formed between the interventions in this study, and the closed loop was tested for consistency. The test of loop inconsistency showed that CT, aerobic exercise, and stretching balance formed a closed loop (IF = 6.70, 95% CI [0, 22.11], *P* = 0.394). Although the point estimates were high, the confidence intervals were wide and included 0, suggesting that statistically significant inconsistencies were not observed with the current evidence. Considering the limited number of relevant studies and small sample size, this result should still be interpreted with caution. Therefore, the subsequent estimation of the combined effect sizes and efficacy ranking using the consistency model are still of some reliability. Details of the results are shown in **Supplementary Table 6**. According to the SUCRA analysis, multicomponent exercise ranked relatively high for the UPDRS III outcome (SUCRA = 87.4%), but the results should be interpreted with caution due to the low confidence of the relevant comparative evidence. The SUCRA values of the remaining interventions are shown in **Figures 4**, **5D**.

### Subgroup analysis by disease type

**Figure 6:**
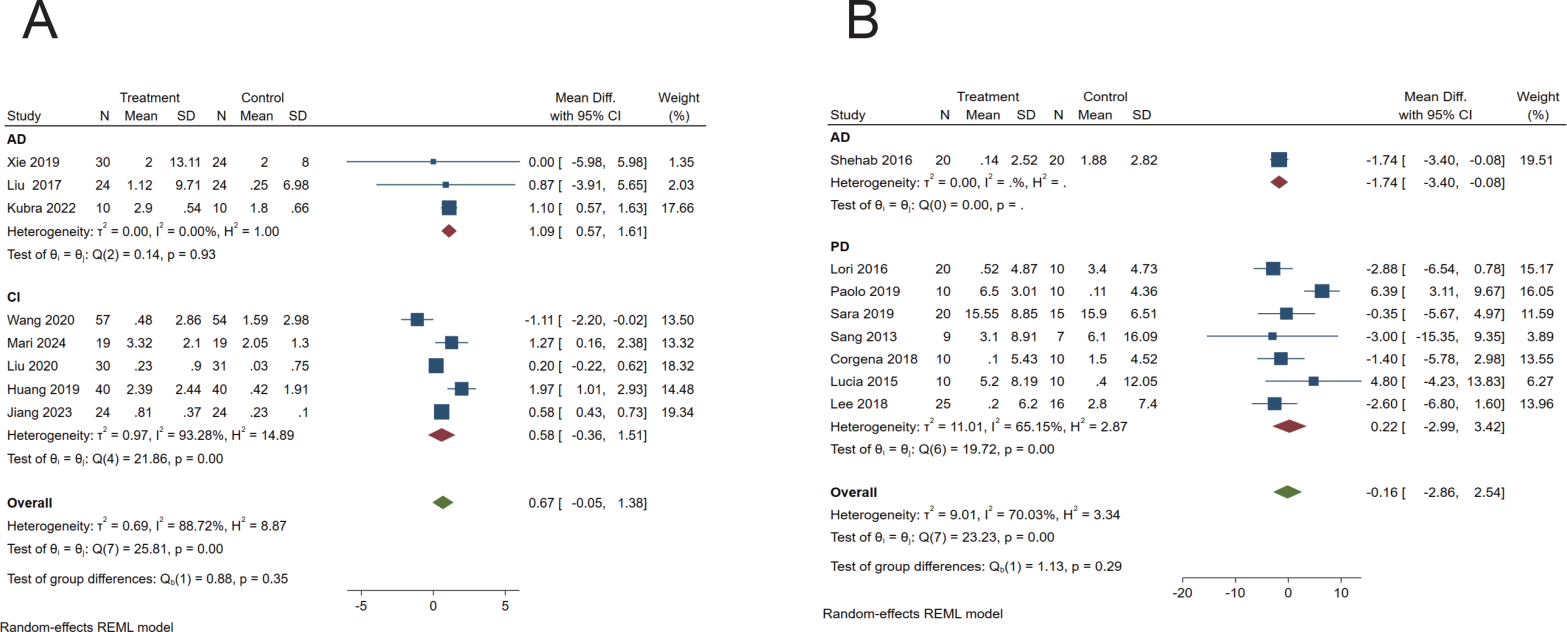
Subgroup analyses by disease type for the primary outcomes. The disease subgroups included Alzheimer’s disease (AD), cognitive impairment, and Parkinson’s disease (PD). **(A)** Geriatric Depression Scale (GDS). **(B)** Beck Depression Inventory (BDI). The forest plots show the pooled mean differences (MDs) and 95% confidence intervals (CIs) for exercise interventions versus control, stratified by disease type. Random-effects models were used for all subgroup analyses.

To further assess the possible clinical heterogeneity by disease type, the present study conducted supplementary subgroup analyses of the primary outcomes by disease type. Subgroup analyses of the GDS outcomes showed a combined effect size of MD = 1.09 (95% CI [0.57, 1.61]) in the Alzheimer’s disease subgroup and MD = 0.58 (95% CI [-0.36, 1.51]) in the cognitive impairment subgroup, with no statistically significant difference tested between groups (*P* = 0.35); the cognitive impairment subgroup was more heterogeneous (*I*² = 93.28%), and the test for between-group differences was not statistically significant. Subgroup analyses of the BDI outcomes showed that only one study was included in the Alzheimer’s disease subgroup, with an effect size of MD = -1.74 (95% CI [-3.40, -0.08]); the combined effect size in the PD subgroup was MD = 0.22 (95% CI [-2.99, 3.42]), and the test for between-group differences was not statistically significant (*P* = 0.29). The results are shown in **Figure 6**. Overall, the current complementary analysis did not suggest clear differences in between-group effects between different disease types, but due to the limited number of studies in some of the subgroups, the relevant results should still be considered as exploratory evidence.

### Risk of publication bias

**Figure 7:**
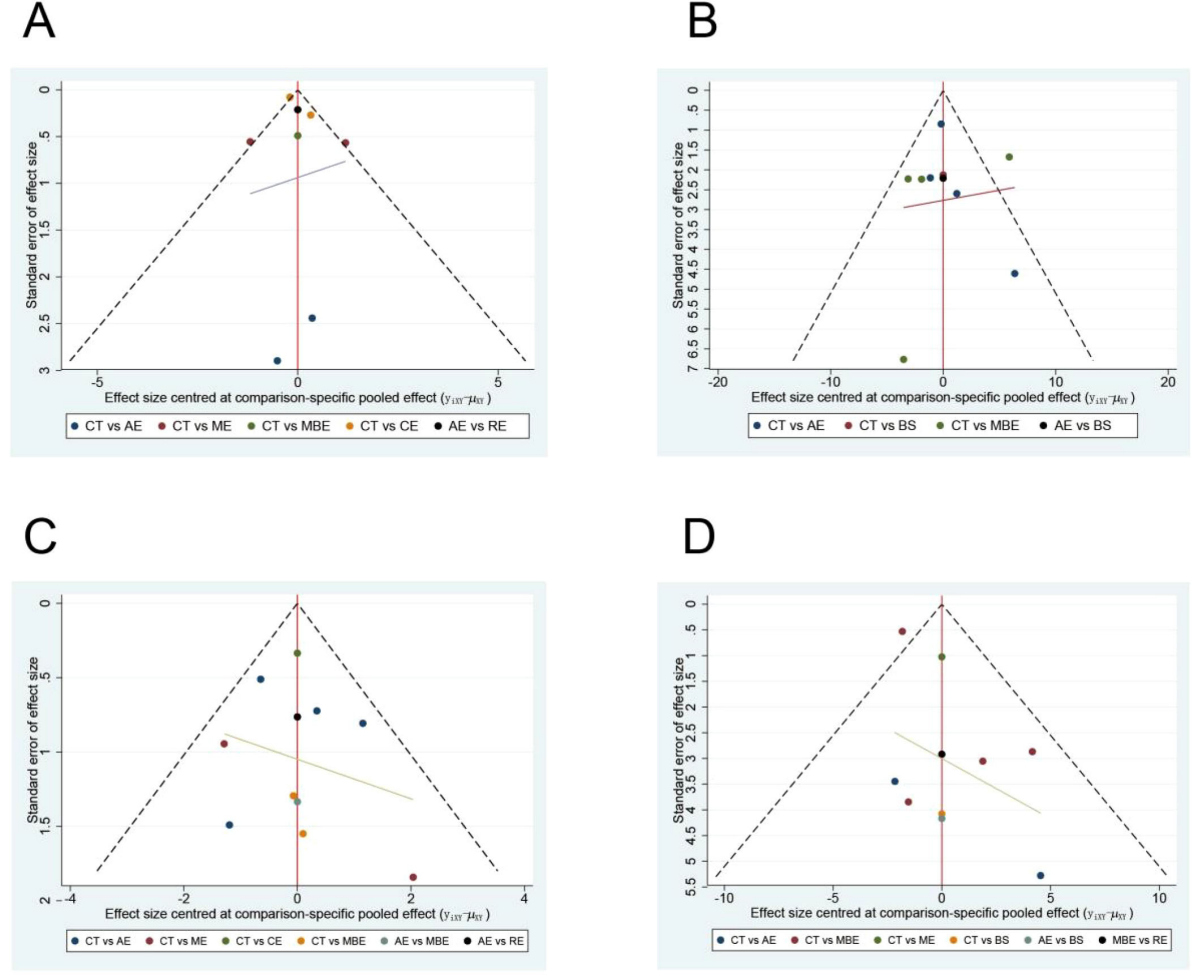
Funnel plot for publication bias in all outcomes. **(A)** GDS, **(B)** BDI, **(C)** MMSE, **(D)** UPDRS III. GDS, Geriatric Depression Scale; BDI, Beck Depression Inventory; MMSE, Mini-Mental State Examination; UPDRS III, Unified Parkinson’s Disease Rating Scale III.

Potential publication bias across outcome-related studies was descriptively assessed using comparison-adjusted funnel plots, and the results are shown in **Figure 7**. In the funnel plots of GDS and BDI, the study sites were generally distributed on both sides of the midline, and the overall symmetry was good, with no obvious signs of publication bias. In contrast, the funnel plots of MMSE showed a certain degree of asymmetry, suggesting that there might be a small-sample effect or potential publication bias, and the funnel plots of UPDRS III also showed a certain degree of asymmetry, but in view of the limited number of included studies, the results should be interpreted with caution.

The asymmetry of the MMSE funnel plot may be related to selective publication bias and small-sample effect. As an important indicator of cognitive function, positive MMSE-related results are more likely to be published, whereas negative studies may be underpublished. Meanwhile, small-sample studies are more susceptible to randomization errors, which may lead to unstable effect size estimates. Therefore, the MMSE-related results should be interpreted with caution and need to be further validated by more high-quality randomized controlled studies.

### Certainty of evidence assessment

The certainty of the evidence was assessed for all pairwise comparisons of the four outcome indicators using the CINeMA framework. The results showed that for the primary outcome, the evidence credibility of most comparisons in GDS and BDI was low, mainly affected by imprecision and heterogeneity, and combined with network meta-analyses no significant differences were observed, and the associated results need to be interpreted with caution. In terms of secondary outcomes, some MMSE comparisons were supported by relatively high-certainty evidence, particularly those supporting the superiority of CE over RE and MBE, which were assessed as “high”. In addition, the structure of the evidence for comparisons between CT and ME, CT and CE, and AE and CE was relatively robust. In contrast, the certainty of evidence for UPDRS-III-related comparisons was generally low. The certainty of evidence for comparisons between CT and AE, BS, and MBE was rated as low. Although the comparison between ME and MBE was statistically significant, the certainty of evidence was rated as very low because of heterogeneity, indicating substantial uncertainty in this finding. Details of the results are shown in **Supplementary Tables 11**-**S14**.

## Discussion

### Summary of key findings

In terms of primary outcomes, the network meta-analyses of the Geriatric Depression Scale (GDS) and the Beck Depression Inventory (BDI) showed that no statistically significant differences were observed between exercise interventions and the control group or among different exercise interventions. This suggests that in the current evidence framework, there is insufficient evidence to support a clear and consistent superiority of a particular exercise modality in improving depressive symptoms in patients with Alzheimer’s disease, cognitive impairment, and Parkinson’s disease. Considering that the included populations involved different disease backgrounds, with possible differences in depressive phenotypes, disease stage, and response to exercise, the aforementioned clinical heterogeneity may dilute the intervention effect to some extent and increase the uncertainty of the network estimation.

The lack of statistically significant differences in the primary outcomes in this study may be related to the following factors. First, the effect of exercise intervention on depressive symptoms may be relatively mild and its effect usually relies on the gradual process of neurotransmitter modulation, inhibition of inflammatory response, and alteration of neuroplasticity, and the follow-up period of most of the included studies was 2–4 months, which may be insufficient to observe significant changes in the scale scores. Second, there were differences in the type of exercise, frequency, intensity, duration, and control measures among the included studies, which may have reduced the statistical power. Third, although GDS and BDI are commonly used depression assessment tools, their applicability in the cognitively impaired population may be limited, especially in some moderately severe patients, and the bias of emotion expression and entry comprehension may affect the sensitivity of the scales.

It should be noted that although the SUCRA ordering can provide a reference for the relative ranking of different intervention modalities, in the absence of statistically significant differences in the primary outcomes, the results of the ordering should only be regarded as exploratory information, and it is not appropriate to infer that a certain exercise modality has a clear clinical superiority. Therefore, the results of the present study support that “there is currently no stable evidence to support clear differences in efficacy among exercise modalities in improving depressive symptoms” and that randomized controlled trials with larger sample sizes, clearer disease stratification, and more standardized exercise prescriptions are needed to further validate the potential effects of different exercise modalities on depressive symptoms. In addition, complementary subgroup analyses did not show clear group differences in the primary outcome across disease subgroups, but the results should be considered as exploratory evidence due to the limited number of studies included in some of the subgroups and the high heterogeneity of the subgroups.

Unlike the primary outcome, the analysis of secondary outcomes suggests that some exercise modalities may have potential benefits in terms of cognitive and motor function, and the MMSE results showed that CE significantly improved the scores compared with CT and was superior to RE, AE, MBE, and ME in some of the indirect comparisons, suggesting that it may have an advantage in terms of cognitive function. However, MMSE was a secondary outcome in this study, and the results may still be affected by the limited number of studies and potential publication bias, so they should be interpreted with caution. The UPDRS III results showed that the overall exercise intervention did not reach statistical significance in improving motor function, but the indirect comparison suggested that multicomponent exercise might be more beneficial than mind–body exercise for improving motor function in patients with Parkinson’s disease. However, the level of evidence for this comparison is very low, and clinical interpretation should be cautious.

Overall, the present study suggests that the potential benefits of exercise interventions in these populations may not only be in terms of depressive symptoms but also in terms of cognitive- and motor-related outcomes. However, as the primary outcome did not show clear statistical superiority and the evidence for secondary outcomes is still limited, the current results are more appropriately viewed as hints and directions for future research.

### Comparison with other studies

Compared with previous studies, the present findings show both similarities and differences. Earlier meta-analyses mainly focused on whether exercise interventions were effective, and most were limited to a single type of physical activity or a single disease population—for example, some meta-analyses showed that resistance training and mind–body exercise improved depressive symptoms in older adults (Tian et al., 2024). Other studies have reported that aerobic exercise significantly improves MMSE scores in patients with Alzheimer’s disease (Zhang et al., 2022), whereas multicomponent exercise has been shown to improve cognitive function, attention, and memory in individuals with mild cognitive impairment (Wang et al., 2024). These studies mainly addressed whether exercise interventions have an overall effect compared with usual care but less often compared the relative efficacy of different exercise modalities within a unified analytical framework.

The present study is characterized by the inclusion of a population with neurodegenerative diseases on the spectrum of Alzheimer’s disease, cognitive impairment, and Parkinson’s disease and by the use of network meta-analysis to simultaneously compare the relative effects of multiple exercise modalities; therefore, the differences between the present study and previous studies may primarily reflect differences in the population, the way in which the interventions were categorized, and the framework of the comparisons.

### Interpretation of findings and possible mechanisms

Although the present study did not observe a clear statistical advantage of different exercise modalities on the improvement of depressive symptoms in the primary outcome, this does not mean that the exercise intervention was completely ineffective in the abovementioned population. The clinical benefits of exercise-related interventions may be multidimensional in nature and may not be characterized by significant changes in depression scale scores but rather by improvements in cognitive functioning, motor functioning, or overall health status.

Previous studies have suggested that exercise may influence symptoms associated with depression and neurodegenerative diseases through multiple biological pathways—for example, exercise may increase the hippocampal levels of serotonin, norepinephrine (NE), and brain-derived neurotrophic factor (BDNF) while reducing central inflammatory responses, thereby potentially contributing to neuroprotection and mood regulation (Ross et al., 2023). In addition, physical activity may protect the aging brain by increasing cerebral blood flow and reducing Aβ formation and tau phosphorylation (De la Rosa et al., 2020). Long-term exercise has also been shown to increase the levels of BDNF and reduce inflammatory markers such as CRP, TNF-α, and IL-6, thereby potentially supporting cognitive function (Yu et al., 2009; De la Rosa et al., 2019). These findings provide a biological basis for the potential of exercise interventions to improve cognitive and mood-related outcomes, although these mechanisms were not directly examined in the present study.

On the other hand, depression has a long and complex association with neurodegenerative diseases and cognitive decline (Fuchs, 2007). Previous studies have suggested that greater depression severity is associated with cognitive decline and that depression in the prodromal stages of dementia, including mild cognitive impairment, is associated with white matter atrophy and an increased risk of subsequent cognitive decline (Jamieson et al., 2019). In this context, the potential value of exercise interventions may lie not only in the direct alleviation of depressive symptoms but also in their indirect effects on cognitive and emotional outcomes through improvements in the neurobiological environment and functional status. This may partly explain why no significant differences were observed in the primary outcome of this study, whereas some secondary outcomes still showed potential benefits.

In patients with Parkinson’s disease, previous studies have suggested that exercise may stabilize motor progression and improve cognitive performance by promoting functional and structural plasticity in cortical sensorimotor and cognitive control networks (Johansson et al., 2022). Multiple systematic reviews have also reported benefits of physical activity for motor symptoms, quality of life, and subjective well-being in patients with Parkinson’s disease (Tomlinson et al., 2013; Ćwiękała-Lewis et al., 2017). However, the overall UPDRS III results in this study did not show a clear statistically significant difference, and only some indirect comparisons suggested that multicomponent exercise training might be more favorable than mind–body exercise for improving motor function; moreover, the certainty of the relevant evidence was low. Therefore, the interpretation of motor benefits in Parkinson’s disease should remain cautious.

Combined with the results of the present study, cognitive exercise showed some potential advantages in MMSE outcomes, suggesting that the benefits of exercise-related interventions may be more likely reflected in cognitive dimensions rather than direct improvements in depression scale scores under the current evidence. Overall, these mechanistic evidences may provide some explanations for the directional results of the secondary endpoints in this study, but they are still insufficient to replace high-quality clinical evidence. Future studies with more standardized design, clearer stratification, and more standardized exercise prescription are still needed to further validate the real roles of different exercise modalities in the outcomes of depression, cognition, and motor function.

### Limitations and strengths

#### Evidence base, heterogeneity, and methodological limitations

The present study has several limitations. First, the overall number of included studies was limited, and some of the intervention comparisons were based on a small number of studies with small sample sizes, which may reduce the precision of effect estimates and affect the stability of the results of the network meta-analysis. Second, this study included three groups of people with Alzheimer’s disease, cognitive impairment, and Parkinson’s disease. Although these diseases are often associated with depressive symptoms, their pathological basis, disease stage, functional impairment characteristics, and responses to exercise interventions may not be identical, which may increase clinical heterogeneity and affect the plausibility of the transitivity assumption. It should be noted that, although the present study, in which mild cognitive impairment and dementia were analyzed under the category of “cognitive impairment”, helped improve study integration, there may still be important differences between the different stages of the disease.

In addition, there were significant differences in the intervention programs of the included studies, including the type of exercise, frequency, intensity, duration, total dose, setting of control measures, etc. There was also a certain degree of heterogeneity within some of the intervention categories, especially cognitive exercise and multicomponent exercise, whose specific training contents and pathways of action were not identical and which may have affected the comparability of the different studies. In terms of methodology, some of the studies were not adequately reported on randomization, allocation concealment, and blinding, suggesting that the risk of intra-study bias still exists. For outcome indicators, the performance of depression scales in the cognitively impaired population may be affected by factors such as comprehension, expression, and care environment, which may increase the uncertainty of outcome assessment. It is worth noting that there was a certain degree of asymmetry in the comparative corrected funnel plots of MMSE-related outcomes, suggesting a small-sample effect or potential publication bias. Therefore, the results of the present study, especially the secondary outcomes and intervention ranking results, should be interpreted with caution and need to be further validated in more randomized controlled trials with clearer stratification, more standardized exercise prescriptions, and larger sample sizes.

#### Risk of publication bias

In the present study, we used comparative corrected funnel plots for the descriptive assessment of the outcome indicators. The results showed that the funnel plots of GDS and BDI, which were the primary outcomes, had good symmetry and showed no obvious signs of publication bias; in contrast, the funnel plots of MMSE showed a certain degree of asymmetry, suggesting that the outcomes related to cognitive functioning might be affected by small-sample effects or potential publication bias. It is important to note that although there was no significant funnel plot abnormality in the depressive symptom-related outcomes, the network comparisons of the GDS and BDI in this study did not show statistically significant differences, in general, and therefore the results of the current evidence need to be interpreted with caution.

#### Strengths

The present study has several advantages. Firstly, this study included three groups of people with Alzheimer’s disease, cognitive impairment, and Parkinson’s disease under a unified analytic framework, focusing on the common and clinically important outcome of depressive symptoms, which better reflects the value of comparative effectiveness of different exercise modalities compared with previous studies focusing on a single disease or a single outcome. Secondly, the present study included 23 randomized controlled trials and systematically compared six types of exercise interventions, focusing not only on the primary outcome of depressive symptoms but also on cognitive function, motor function, and other related outcomes so as to evaluate the potential impact of exercise interventions in a more comprehensive manner. Thirdly, this study strictly followed the PRISMA reporting standard and was registered on the PROSPERO platform (no. CRD420261280926), and this adopted a two-investigator independent screening of the literature, extraction of data, and evaluation of the risk of bias to minimize selection bias and information bias in the research process. Finally, this study used a network meta-analysis method, based on the integration of direct and indirect evidence, to compare multiple exercise modalities in a unified manner and provide exploratory information on the relative ranking of interventions by SUCRA ranking, which can provide some references for subsequent studies and clinical individualized exercise prescription.

### Clinical advice and recommendations

Based on the current evidence, there is insufficient evidence to support clear recommendations regarding the prioritization of specific exercise modalities. In clinical practice, exercise interventions can be individualized according to the patient’s disease type, functional status, exercise capacity, adherence, and safety and can be used as an adjunct to conventional treatment. For secondary outcomes such as cognitive function and motor function, some exercise modalities show potential benefits, but the evidence is still limited and clinical interpretation should be cautious.

Future studies should prioritize randomized controlled trials with larger sample sizes, clearer disease stratification, and more standardized intervention protocols, focusing on assessing the long-term effects of different exercise modalities on depressive symptoms, cognitive function, and motor function and further clarifying the applicable populations, the dose–response relationship, and the potential mechanism of action of different exercise prescriptions. An appropriate extension of the follow-up period will also help to assess the sustained effect and clinical value of the exercise intervention more comprehensively.

## Conclusion

This study compared the effects of different exercise interventions on depressive symptoms in patients with Alzheimer’s disease, cognitive impairment, and Parkinson’s disease by network meta-analysis. The results showed that no statistically significant differences were observed in the overall improvement of depressive symptoms by different exercise modalities, and there is insufficient evidence to support a clear and consistent superiority of a particular exercise modality in the improvement of depressive symptoms. Although some of the secondary outcomes suggested that cognitive exercise and multicomponent exercise training might have some potential benefits, the results should be interpreted with caution.

## Data Availability

The original contributions presented in the study are included in the article/**Supplementary Material**. Further inquiries can be directed to the corresponding author.
