# Supplementary material for: Comparative efficacy of exercise interventions on depressive symptoms and related outcomes in patients with Alzheimer’s disease, cognitive impairment, and Parkinson’s disease: a systematic review and network meta-analysis
Source: Front Physiol. 2026 May 25;17:1825740. doi: 10.3389/fphys.2026.1825740 (PMC13243066; doi:10.3389/fphys.2026.1825740)
Supplement: Supplementary file 1 [file DataSheet1.docx]

Supplementary Table S1 Study characteristics relevant to transitivity and intervention comparability

| Paper | Disease population | Intervention category | Specific intervention | Baseline depression severity / scale | Duration (weeks) | Frequency (sessions/week) | Intensity | Total weekly dose | Comparators | Comments relevant to transitivity |
| --- | --- | --- | --- | --- | --- | --- | --- | --- | --- | --- |
| Xie 2019 | Alzheimer’s disease | AE | Moderate-intensity aerobic exercise | Geriatric Depression Scale, median 10 (exercise) vs 13 (control) | 12 | 3 | 70% of maximum heart rate | 120 min/week | Health education only | MMSE<25; age 50–85 years; Hachinski Ischemic Scale ≤4 |
| Liu 2020 | Dementia | AE | Stationary bicycle training | Geriatric Depression Scale-5, mean 0.48 (aerobic) vs 0.40 (strength) | 4 | 5 | Rated perceived exertion 5–6/10; 30 min/session | 150 min/week | RE | MMSE15-26;nursing home residents |
| Liu 2017 | Alzheimer’s disease | AE | Moderate-intensity aerobic exercise training | Geriatric Depression Scale, mean 11.13 (exercise) vs 11.25 (control) | 12 | 3 | 70% of maximum heart rate | 120 min/week | Usual daily activity without aerobic training | MMSE<25;age 50–80 years; Hachinski Ischemic Scale ≤4 |
| Kristine 2015 | Alzheimer’s disease | AE | Supervised moderate-to-high intensity aerobic exercise | Hamilton Depression Rating Scale-17, mean 1.9 (intervention) vs 2.0 (control) | 16 | 3 | 70–80% of maximal heart rate | 180 min/week | Treatment as usual | MMSE＞19；community-dwelling patients with mild Alzheimer’s disease |
| Sara 2019 | Parkinson’s disease (idiopathic) | AE | High cadence cycling on a motorized stationary ergometer | Beck Depression Inventory-II, mean 9.45 (cycling) vs 9.7 (control) | 1 | 3 | Low-to-moderate intensity; approximately 50–60% of age-predicted maximal heart rate | 120 min/week | No cycling control (maintained normal activity) | Montreal Cognitive Assessment mean 25.7; age 50–85 years; no surgical procedures for Parkinson’s disease |
| Li 2012 | Alzheimer’s disease | AE | Running | Hamilton Rating Scale for Depression, baseline 6.23 | 24 | 6 | 1 h/session; intensity not clearly reported | 360 min/week | MBE、Control group (no regular systematic physical exercise) | Multi-arm trial; CDR 1–3; MMSE mean =17.90 at baseline |
| Zhou 2024 | Post-stroke dementia | AE | Progressive resistance stationary cycling | Hamilton Depression Scale, mean 12.70 (aerobic exercise) vs 12.25 (control) | 8 | 5 | Target heart rate 40%–70% of maximum heart rate | 150 min/week | Routine treatment only | able to perform aerobic exercise; with or without assistive walking devices |
| Shehab 2016 | Alzheimer’s disease | AE | Treadmill aerobic exercise | Beck Depression Inventory, mean 8.01 (exercise) vs 7.82 (control) | 8 | 3 | 60–70% of maximum heart rate | 30–90 min/week | No training intervention | Supervised by physical therapist; stable ordinary diet |
| Lucia 2015 | Parkinson’s disease (Hoehn and Yahr 1–3 | AE | Nordic Walking | BDI-II, mean 14.8 (NW) vs 14.4 (control) | 12 | 2 | 60–80% heart rate reserve | 120 min/week | Conventional care | MMSE ≥24; stable medication use |
| Lori 2016 | Parkinson’s disease (idiopathic PD), H&Y stage 1–3 | AE | Treadmill aerobic exercise | BDI, mean 5.4 (AE) vs 6.1 (BS) vs 4.2 (CT) | 16 | 3 | 50% to 75% heart rate reserve (progressively increased) | 60–135 min/week | BS; no-contact control (CT) | Three-arm trial; **MMSE ≥25** |
| Wang 2020 | Mild cognitive impairment | ME | Structured limbs-exercise program | GDS, mean 5.59 (intervention) vs 5.15 (wait-list control) | 24 | 3 | 60–80% of maximum heart rate | 180 min/week | Health promotion classes alone (wait-list control) | MMSE25-30;Montreal Cognitive Assessment ≤26 |
| MARI 2024 | Dementia (early- to middle-stage) | ME | Structured group exercise program (strengthening and balance exercises) | Geriatric Depression Scale, mean 5.89 (intervention) vs 2.95 (control) | 36 | 3 | Progressive increase from 1 set of 8 repetitions to 3 sets of 12 repetitions | 150 min/week | Usual medical care and light recreational activities | Supervised group exercise; Mini-Mental State Examination 10–24 |
| Marinda 2018 | Dementia (nursing home residents; MMSE 1–24) | ME | Multicomponent exercise training (alternating strength and aerobic sessions) | Cornell Scale for Depression in Dementia, exercise group 8.3 vs control group 8.1 | 24 | 3 | Progressive increase in intensity; intended 3 sets of 8 repetitions, progressing to 10–15 repetitions and additional weights; aerobic walking 500 m or 1 km with interval increases | 90–135 min/week | Social activity control (tea with nursing staff) | Group-based; moderate adherence (55%); intensity not always achieved as intended |
| Wu 2021 | Parkinson’s disease (Hoehn and Yahr stage I–II) | ME | Home-based aerobic and resistance exercise | Geriatric Depression Scale short form, mean 5.20 (exercise) vs 6.12 (control) | 8 | 3-5 | 60–80% of maximum heart rate | 120–150 min/week | Regular lifestyle (no exercise) | MMSE≥16; stable medication for at least 3 months |
| Danny 2016 | Parkinson’s disease (idiopathic PD; H＆Y1–3) | MBE | Iyengar yoga | BDI, mean 4.57 (yoga) vs 7.57 (resistance) | 12 | 2 | Not clearly reported | 120 min/week | RE | Mild–moderate PD; MoCA ≥24 |
| Huang 2019 | Mild dementia | MBE | Modified Tai Chi program | GDS mean 4.86 (Tai Chi) vs 4.94 (control) | 40 | 3 | 20 min/session; moderate intensity not further quantified | 60 min/week | Routine treatments and personalized daily care | Montreal Cognitive Assessment mean 13.08; Clinical Dementia Rating <2 |
| Paolo 2019 | Parkinson’s disease(Hoehn and Yahr ≤3) | MBE | Sardinian folk dance (Ballu Sardu) | BDI,mean 14.10 (Ballu Sardu) vs 13.67 (control) | 12 | 2 | 90 min/session; intensity not clearly quantified | 180 min/week | Usual care alone | MMSE≥24 |
| Sang 2013 | Parkinson’s disease (mild-to-moderate) | MBE | Tai Chi (Tai-Chi-for-arthritis program, 12 Sun-style movements) | Beck’s Depression Inventory, mean 23.4 (Tai Chi) vs 23.2 (control) | 8 | 3 | Perceived exertion not clearly reported for Tai Chi | Not clearly reported | Nonintervention control | Female participants only in analysis; stable medication for >6 months |
| Corgena 2018 | Parkinson’s disease (H＆Y I–III) | MBE | Hatha yoga | BDI, mean 8.8 (yoga) vs 7.1 (wait-list control) | 12 | 2 | Not clearly reported | 120 min/week | Wait-list control | Mild-moderate PD; MoCA ≥26 |
| Lee 2018 | Parkinson’s disease (Hoehn and Yahr 1–3) | MBE | Turo (Qi dance) program | BDI, mean 11.2 (Turo) vs 13.3(waiting-list control) | 8 | 2 | 60-min sessions | 120 min/week | Waiting-list control | K-MMSE >20; no exercise therapy within prior 3 months |
| Zhu 2019 | Parkinson’s disease ( Hoehn and Yahr stage I–III) | MBE | Simplified Tai Chi plus routine exercise | Hamilton Depression Scale, mean 16.95 (Tai Chi) vs 16.47 (control) | 12 | 5 | 40–50 min/session; intensity not clearly quantified | 200–250 min/week | Routine exercise alone | Montreal Cognitive Assessment Beijing version ≥17 |
| Kubra 2022 | Alzheimer’s disease | CE | Motor-cognitive dual-task exercise via telerehabilitation | Geriatric Depression Scale-Short Form, mean 6.2 (telerehabilitation) vs 4.2 (control) | 6 | 4-5 | Progressive chair-based real-time supervised exercise; intensity not clearly quantified | 75–160 min/week | No intervention | Mini-Mental State Examination 13–24; Clinical Dementia Rating 1–2 |
| Jiang 2023 | Mild cognitive impairment | CE | Cup-stacking training | 30-Geriatric Depression Scale, mean 6.23 (intervention) vs 5.48 (control) | 24 | ≥5 | ≥30 min/session; intensity not clearly quantified | ≥150 min/week | Routine outpatient management | Montreal Cognitive Assessment indicated mild cognitive impairment; 6-month home-based progressive training |

Supplementary Table S2 Basic information of all the papers

| Paper | Diagnostic criteria | Sample size (Control group/Treatment group/Other groups) | Gender  (M/F) | Control group | Treatment group | Other group | Intervention  Control group | Intervention  Treatment group | Intervention  Other group | age(years)  Control group | age(years)  Treatment group | age(years)  Other group | Duration of treatment(month） | Outcomes | randomization method |
| --- | --- | --- | --- | --- | --- | --- | --- | --- | --- | --- | --- | --- | --- | --- | --- |
| Xie 2019 | MMSE<25 | 24/30 | 27/27 | 12/12 | 15/15 | _ | CT | AE | _ | 72.7±7.6 | 72.0±9.1 | _ | 3 | ①③ | Random |
| Liu  2017 | MMSE<25 | 24/24 | 21/27 | 11/13 | 10/14 | _ | CT | AE | _ | 70.3±7.7 | 70.9±9.2 | _ | 3 | ①③ | Random |
| Wang 2020 | MMSE 20-25、MocA≤26 | 54/57 | 43/68 | 22/32 | 21/36 | _ | CT | ME | _ | 68.24±5.15 | 68.37±5.27 | _ | 6 | ① | Automated Online Randomization Program |
| MARI  2024 | MMSE 10-24 | 19/19 | 31/7 | 14/5 | 17/2 | _ | CT | ME | _ | 80.1±7.2 | 81±6.8 | _ | 9 | ①③ | Random |
| Kubra  2022 | MMSE 13-24  CDR 1-2 | 10/10 | 6/14 | 3/7 | 3/7 | _ | CT | CE | _ | 80.6±6.11 | 77.7±5.29 | _ | 1.5 | ①③ | Random Number Table |
| Liu  2020 | MMSE 15-26 | 31/30 | 50/11 | 26/5 | 24/6 | _ | AE | RE | _ | 84.68±6.74 | 86.77±6.99 | _ | 1 | ①③ | flip a coin |
| Lori  2016 | Diagnosed as PD,H&Y’s stage 1-3 | 10/9/11 | _ | _ | _ | _ | CT | BS | AE | 67.8±9.8 | 63.3±7.3 | 62.8±8.6 | 4 | ②③④ | Random |
| Kristine  2015 | AD、MMSE≥19 | 93/107 | 113/87 | 57/36 | 56/51 | _ | CT | AE | _ | 71.3±7.3 | 69.8±7.4 | _ | 4 | ③ | Blocked Randomization |
| Huang  2019 | Diagnosed as AD | 40/40 | 26/54 | 14/26 | 12/28 | _ | CT | MBE | _ | 81.9±6.1 | 81.9±6.0 | _ | 10 | ①③ | Automated Online Randomization Program |
| Paolo  2019 | H＆Y≤3、MMSE≥24 | 10/10 | 13/7 | 7/3 | 6/4 | _ | CT | MBE | _ | 67.1±6.3 | 67.8±5.9 | _ | 3 | ②④ | Automated Online Randomization Program |
| Sara  2019 | Diagnosed as PD | 15/20 | 23/12 | 12/3 | 11/9 | _ | CT | AE | _ | 64.87 ± 6.90 | 65.05 ± 9.13 | _ | 0.25 | ② | Unclear |
| Jiang  2023 | MoCA≤24 | 24/24 | 21/27 | 11/13 | 10/14 | _ | CT | CE | _ | 72.67 +±5.32 | 74.08±6.12 | _ | 6 | ① | Automated Online Randomization Program |
| Li  2012 | Diagnosed as AD | 40/40/40 | 65/95 | _ | _ | _ | CT | AE | MBE | _ | _ | _ | 6 | ③ | Random |
| Zhou  2024 | MMSE≤23 | 20/20 | 24/16 | 12/8 | 12/8 | _ | CT | AE | _ | 58.70±8.97 | 58.70±9.47 | _ | 2 | ③ | Automated Online Randomization Program |
| Marinda  2018 | MMSE1-24 | 22/22 | 10/34 | 5/17 | 5/17 | _ | CT | ME | _ | 84.73±4.55 | 85.14±4.64 | _ | 6 | ③ | Random Number Table |
| Sang  2013 | Diagnosed as PD | 7/9 | _ | _ | _ | _ | CT | MBE | _ | 64.9±7.2 | 65.6±7.9 | _ | 2 | ②④ | Unclear |
| Shehab  2016 | Diagnosed as AD | 20/20 | 29/11 | 15/5 | 14/6 | _ | CT | AE | _ | 69.13±6.12 | 68.94±5.76 | _ | 2 | ② | Blocked Randomization |
| Danny  2016 | Diagnosed as PD,H&Y1-3 | 7/7 | 11/3 | 6/1 | 5/2 | _ | MBE | RE | _ | 66.7 ± 9.3 | 67.9±10.9 | _ | 3 | ③ | Blocked Randomization |
| Corgena  2018 | Diagnosed as PD,H&Y1-3 | 10/10 | 15/11 | 8/5 | 7/6 | _ | CT | MBE | _ | 65.8 ± 6.6 | 63.5±8.5 | _ | 3 | ②④ | Automated Online Randomization Program |
| Lucia  2015 | Diagnosed as PD,H&Y1-3 | 10/10 | _ | _ | _ | _ | CT | AE | _ | 66.6±7.3 | 68.1±8.7 | _ | 3 | ②④ | Random |
| Wu  2021 | Diagnosed as PD,H&Y1-2  MMSE≥16 | 49/49 | 56/42 | 30/19 | 26/23 | _ | CT | ME | _ | 66.59±8.61 | 63.65±6.02 | _ | 2 | ①④ | Automated Online Randomization Program |
| Lee  2018 | Diagnosed as PD,H&Y1-2 | 16/25 | 17/24 | 7/9 | 10/15 | _ | CT | MBE | _ | 65.7 ± 6.4 | 65.8±7.2 | _ | 2 | ②④ | Blocked Randomization |
| Zhu  2019 | Diagnosed as PD,H&Y1-3 | 22/19 | _ | _ | _ | _ | CT | MBE | _ | 67.77±1.72 | 68.53±1.90 | _ | 3 | ④ | Automated Online Randomization Program |

Note:CT:Conventional Therapy.AE:Aerobic Exercise.RE:Resistance Exercise.BS:Balance Stretching.MBE:Mind-Body Exercise.ME:Multicomponent Exercise.CE:Cognitive Exercise.①GDS;②BDI;③MMSE;④UPDRSⅢ

| Interventions | CT | ME | AE | RE | CE | MBE |
| --- | --- | --- | --- | --- | --- | --- |
| MBE | -1.97 (-4.10,0.16) | -1.90 (-4.53,0.74) | -1.47 (-5.92,2.98) | -1.27 (-6.13,3.59) | -1.14 (-3.67,1.40) | 0 |
| CE | -0.83 (-2.20,0.54) | -0.76 (-2.83,1.32) | -0.33 (-4.47,3.81) | -0.13 (-4.71,4.44) | 0 |  |
| RE | -0.70 (-5.06,3.66) | -0.63 (-5.26,4.01) | -0.20 (-2.15,1.75) | 0 |  |  |
| AE | -0.50 (-4.40,3.40) | -0.43 (-4.63,3.78) | 0 |  |  |  |
| ME | -0.07 (-1.63,1.48) | 0 |  |  |  |  |
| CT | 0 |  |  |  |  |  |

Supplementary Table S3 Results of network Meta-Analysis(GDS)

Supplementary Table S4 Results of network Meta-Analysis(BDI)

| Interventions | BS | AE | CT | MBE |
| --- | --- | --- | --- | --- |
| MBE | -3.01 (-10.77,4.74) | -1.67 (-7.32,3.97) | -0.81 (-4.84,3.23) | 0 |
| CT | -2.21 (-8.80,4.38) | -0.87 (-4.71,2.97) | 0 |  |
| AE | -1.34 (-7.96,5.28) | 0 |  |  |
| BS | 0 |  |  |  |

Supplementary Table S5 Results of network Meta-Analysis(MMSE)

| Interventions | CE | AE | RE | CT | MBE | ME |
| --- | --- | --- | --- | --- | --- | --- |
| ME | **6.30 (3.94,8.66)** | **3.32 (1.16,5.47)** | 2.82 (-0.05,5.69) | **2.80 (0.85,4.75)** | 2.41 (-0.33,5.15) | 0 |
| MBE | **3.89 (1.55,6.24)** | 0.91 (-1.10,2.91) | 0.41 (-2.35,3.17) | 0.39 (-1.54,2.32) | 0 |  |
| CT | **3.50 (2.17,4.83)** | 0.52 (-0.44,1.47) | 0.02 (-2.10,2.14) | 0 |  |  |
| RE | **3.48 (0.98,5.99)** | 0.50 (-1.39,2.39) | 0 |  |  |  |
| AE | **2.98 (1.35,4.62)** | 0 |  |  |  |  |
| CE | 0 |  |  |  |  |  |

Supplementary Table S6 Results of network Meta-Analysis(UPDRSⅢ)

| Interventions | ME | CT | RE | AE | BS | MBE |
| --- | --- | --- | --- | --- | --- | --- |
| MBE | **-5.89 (-11.67,-0.11)** | -2.55 (-6.00,0.89) | -2.60 (-9.69,4.49) | -1.50 (-8.72,5.73) | -0.48 (-9.83,8.88) | 0 |
| BS | -5.42 (-15.33,4.49) | -2.08 (-10.83,6.68) | -2.12 (-13.86,9.61) | -1.02 (-9.88,7.84) | 0 |  |
| AE | -4.39 (-12.38,3.59) | -1.05 (-7.55,5.45) | -1.10 (-11.22,9.02) | 0 |  |  |
| RE | -3.29 (-12.44,5.85) | 0.05 (-7.83,7.93) | 0 |  |  |  |
| CT | -3.34 (-7.98,1.30) | 0 |  |  |  |  |
| ME | 0 |  |  |  |  |  |

Note:CT: Conventional Therapy.AE:Aerobic Exercise.RE:Resistance Exercise.ME:Multicomponent Exercise.MBE:Mind-Body Exercise.BS:Balance stretching.CE:Cognitive Exercise. The bold part represents statistical significance.

Supplementary Table S7 League table of the network meta-analysis results(GDS)

| CT |  |  |  |  |  |
| --- | --- | --- | --- | --- | --- |
| -0.07 (-1.63,1.48) | ME |  |  |  |  |
| -0.50 (-4.40,3.40) | -0.43 (-4.63,3.78) | AE |  |  |  |
| -0.70 (-5.06,3.66) | -0.63 (-5.26,4.01) | -0.20 (-2.15,1.75) | RE |  |  |
| -0.83 (-2.20,0.54) | -0.76 (-2.83,1.32) | -0.33 (-4.47,3.81) | -0.13 (-4.71,4.44) | CE |  |
| -1.97 (-4.10,0.16) | -1.90 (-4.53,0.74) | -1.47 (-5.92,2.98) | -1.27 (-6.13,3.59) | -1.14 (-3.67,1.40) | MBE |

Results from the network meta-analysis(mixed [network] and indirect comparisons) are presented in the lower left triangle.Green background color means statistically significant.Note:CT:Conventional Therapy.AE:Aerobic Exercise.MBE:Mind-Body Exercise.ME:Multicomponent Exercise.RE:Resistance Exercise.CE:Cognitive Exercise.

Supplementary Table S8 League table of the network meta-analysis results(BDI)

| BS |  |  |  |
| --- | --- | --- | --- |
| -1.34 (-7.96,5.28) | AE |  |  |
| -2.21 (-8.80,4.38) | -0.87 (-4.71,2.97) | CT |  |
| -3.01 (-10.77,4.74) | -1.67 (-7.32,3.97) | -0.81 (-4.84,3.23) | MBE |

Results from the network meta-analysis(mixed [network] and indirect comparisons) are presented in the lower left triangle.Green background color means statistically significant.Note:CT:Conventional Therapy.AE:Aerobic Exercise.BS:Balance Stretching.MBE:Mind-Body Exercise.

Supplementary Table S9 League table of the network meta-analysis results(MMSE)

| CE |  |  |  |  |  |
| --- | --- | --- | --- | --- | --- |
| **2.98 (1.35,4.62)** | AE |  |  |  |  |
| **3.48 (0.98,5.99)** | 0.50 (-1.39,2.39) | RE |  |  |  |
| **3.50 (2.17,4.83)** | 0.52 (-0.44,1.47) | 0.02 (-2.10,2.14) | CT |  |  |
| **3.89 (1.55,6.24)** | 0.91 (-1.10,2.91) | 0.41 (-2.35,3.17) | 0.39 (-1.54,2.32) | MBE |  |
| **6.30 (3.94,8.66)** | **3.32 (1.16,5.47)** | 2.82 (-0.05,5.69) | **2.80 (0.85,4.75)** | 2.41 (-0.33,5.15) | ME |

Results from the network meta-analysis(mixed [network] and indirect comparisons) are presented in the lower left triangle.Green background color means statistically significant.Note:CT:Conventional Therapy.AE:Aerobic Exercise.MBE:Mind-Body Exercise.ME:Multicomponent Exercise.RE:Resistance Exercise.CE:Cognitive Exercise.

| ME |  |  |  |  |  |
| --- | --- | --- | --- | --- | --- |
| -3.34 (-7.98,1.30) | CT |  |  |  |  |
| -3.29 (-12.44,5.85) | 0.05 (-7.83,7.93) | RE |  |  |  |
| -4.39 (-12.38,3.59) | -1.05 (-7.55,5.45) | -1.10 (-11.22,9.02) | AE |  |  |
| -5.42 (-15.33,4.49) | -2.08 (-10.83,6.68) | -2.12 (-13.86,9.61) | -1.02 (-9.88,7.84) | BS |  |
| **-5.89 (-11.67,-0.11)** | -2.55 (-6.00,0.89) | -2.60 (-9.69,4.49) | -1.50 (-8.72,5.73) | -0.48 (-9.83,8.88) | MBE |

Supplementary Table S10 League table of the network meta-analysis results(UPDRSⅢ)

Results from the network meta-analysis(mixed [network] and indirect comparisons) are presented in the lower left triangle.Green background color means statistically significant.Note:CT:Conventional Therapy.AE:Aerobic Exercise.MBE:Mind-Body Exercise.ME:Multicomponent Exercise.RE:Resistance Exercise.BS:Balance Stretching.

Figure S1 Risk of bias assessment included in the study.


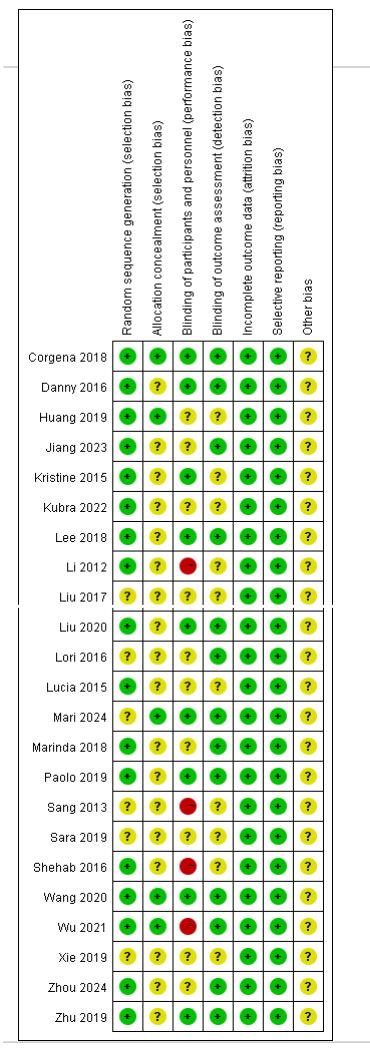


Supplementary Table S11 The Grading of Recommendation Assessment, Development and Evaluation (GRADE) assessment for GDS.

| **Comparison** | **Number of studies** | **Within-study bias** | **Reporting bias** | **Indirectness** | **Imprecision** | **Heterogeneity** | **Incoherence** | **Confidence rating** |
| --- | --- | --- | --- | --- | --- | --- | --- | --- |
| CT:AE | 2 | No concerns | Low risk | No concerns | Major concerns | No concerns | Major concerns | Low |
| CT:ME | 2 | No concerns | Low risk | No concerns | Major concerns | No concerns | Major concerns | Low |
| CT:MBE | 1 | No concerns | Low risk | No concerns | No concerns | Major concerns | Major concerns | Low |
| CT:CE | 2 | No concerns | Low risk | No concerns | Major concerns | No concerns | Major concerns | Low |
| AE:RE | 1 | No concerns | Low risk | No concerns | Major concerns | No concerns | Major concerns | Low |
| CT:RE | 0 | No concerns | Low risk | No concerns | Major concerns | No concerns | Major concerns | Low |
| AE:ME | 0 | No concerns | Low risk | No concerns | Major concerns | No concerns | Major concerns | Low |
| AE:MBE | 0 | No concerns | Low risk | No concerns | Major concerns | No concerns | Major concerns | Low |
| AE:CE | 0 | No concerns | Low risk | No concerns | Major concerns | No concerns | Major concerns | Low |
| ME:RE | 0 | No concerns | Low risk | No concerns | Major concerns | No concerns | Major concerns | Low |
| ME:MBE | 0 | No concerns | Low risk | No concerns | No concerns | Major concerns | Major concerns | Low |
| ME:CE | 0 | No concerns | Low risk | No concerns | Major concerns | No concerns | Major concerns | Low |
| RE:MBE | 0 | No concerns | Low risk | No concerns | Major concerns | No concerns | Major concerns | Low |
| RE:CE | 0 | No concerns | Low risk | No concerns | Major concerns | No concerns | Major concerns | Low |
| MBE:CE | 0 | No concerns | Low risk | No concerns | Major concerns | No concerns | Major concerns | Low |

Supplementary Table S12 The Grading of Recommendation Assessment, Development and Evaluation (GRADE) assessment for BDI.

| **Comparison** | **Number of studies** | **Within-study bias** | **Reporting bias** | **Indirectness** | **Imprecision** | **Heterogeneity** | **Incoherence** | **Confidence rating** |
| --- | --- | --- | --- | --- | --- | --- | --- | --- |
| CT:AE | 4 | No concerns | Low risk | No concerns | Major concerns | No concerns | No concerns | Low |
| CT:BS | 1 | No concerns | Low risk | No concerns | Major concerns | No concerns | No concerns | Low |
| CT:MBE | 4 | No concerns | Low risk | No concerns | Major concerns | No concerns | No concerns | Low |
| AE:BS | 1 | No concerns | Low risk | No concerns | Major concerns | No concerns | No concerns | Low |
| AE:MBE | 0 | No concerns | Low risk | No concerns | Major concerns | No concerns | No concerns | Low |
| BS:MBE | 0 | No concerns | Low risk | No concerns | Major concerns | No concerns | No concerns | Low |

Supplementary Table S13 The Grading of Recommendation Assessment, Development and Evaluation (GRADE) assessment for MMSE.

| **Comparison** | **Number of studies** | **Within-study bias** | **Reporting bias** | **Indirectness** | **Imprecision** | **Heterogeneity** | **Incoherence** | **Confidence rating** |
| --- | --- | --- | --- | --- | --- | --- | --- | --- |
| CT:AE | 4 | No concerns | Low risk | No concerns | Major concerns | No concerns | No concerns | Low |
| CT:ME | 2 | No concerns | Low risk | No concerns | No concerns | No concerns | No concerns | High |
| CT:CE | 1 | No concerns | Low risk | No concerns | No concerns | No concerns | No concerns | High |
| CT:MBE | 2 | No concerns | Low risk | No concerns | Major concerns | No concerns | No concerns | Low |
| AE:RE | 1 | No concerns | Low risk | No concerns | Major concerns | No concerns | No concerns | Low |
| AE:MBE | 1 | Some concerns | Low risk | No concerns | Major concerns | No concerns | No concerns | Very low |
| CT:RE | 0 | No concerns | Low risk | No concerns | Major concerns | No concerns | No concerns | Low |
| AE:ME | 0 | No concerns | Low risk | No concerns | No concerns | No concerns | No concerns | High |
| AE:CE | 0 | No concerns | Low risk | No concerns | No concerns | No concerns | No concerns | High |
| ME:CE | 0 | No concerns | Low risk | No concerns | No concerns | No concerns | No concerns | High |
| ME:RE | 0 | No concerns | Low risk | No concerns | No concerns | Major concerns | No concerns | Low |
| ME:MBE | 0 | No concerns | Low risk | No concerns | Major concerns | No concerns | No concerns | Low |
| CE:RE | 0 | No concerns | Low risk | No concerns | No concerns | No concerns | No concerns | High |
| CE:MBE | 0 | No concerns | Low risk | No concerns | No concerns | No concerns | No concerns | High |
| RE:MBE | 0 | No concerns | Low risk | No concerns | Major concerns | No concerns | No concerns | Low |

Supplementary Table S14 The Grading of Recommendation Assessment, Development and Evaluation (GRADE) assessment for UPDRSⅢ.

| **Comparison** | **Number of studies** | **Within-study bias** | **Reporting bias** | **Indirectness** | **Imprecision** | **Heterogeneity** | **Incoherence** | **Confidence rating** |
| --- | --- | --- | --- | --- | --- | --- | --- | --- |
| CT:AE | 2 | No concerns | Low risk | No concerns | Major concerns | No concerns | No concerns | Low |
| CT:BS | 1 | No concerns | Low risk | No concerns | Major concerns | No concerns | No concerns | Low |
| CT:MBE | 4 | No concerns | Low risk | No concerns | Major concerns | No concerns | No concerns | Low |
| CT:ME | 1 | Some concerns | Low risk | No concerns | Major concerns | No concerns | No concerns | Very low |
| AE:BS | 1 | No concerns | Low risk | No concerns | Major concerns | No concerns | No concerns | Low |
| MBE:RE | 1 | No concerns | Low risk | No concerns | Major concerns | No concerns | No concerns | Low |
| CT:RE | 0 | No concerns | Low risk | No concerns | Major concerns | No concerns | No concerns | Low |
| AE:MBE | 0 | No concerns | Low risk | No concerns | Major concerns | No concerns | No concerns | Low |
| AE:RE | 0 | No concerns | Low risk | No concerns | Major concerns | No concerns | No concerns | Low |
| AE:ME | 0 | No concerns | Low risk | No concerns | Major concerns | No concerns | No concerns | Low |
| BS:MBE | 0 | No concerns | Low risk | No concerns | Major concerns | No concerns | No concerns | Low |
| BS:RE | 0 | No concerns | Low risk | No concerns | Major concerns | No concerns | No concerns | Low |
| BS:ME | 0 | No concerns | Low risk | No concerns | Major concerns | No concerns | No concerns | Low |
| MBE:ME | 0 | Some concerns | Low risk | No concerns | No concerns | Major concerns | No concerns | Very low |
| RE:ME | 0 | No concerns | Low risk | No concerns | Major concerns | No concerns | No concerns | Low |

Within the CINEMA assessment, evidence confidence is classified into four levels. Low risk denotes robust evidence with minimal methodological limitations. Some concerns represents evidence with minor issues that do not seriously affect overall conclusions. High risk indicates serious concerns regarding study design or reporting that may substantially impact the validity of the findings. Unclear is assigned when the available information is inadequate to determine the level of confidence.Note:CT:Conventional Therapy.AE:Aerobic Exercise.MBE:Mind-Body Exercise.ME:Multicomponent Exercise.RE:Resistance Exercise.BS:Balance Stretching.

Appendix S1

Search Strategy

CNKI

SU=（运动 + 拉伸 + 伸展运动 + 水疗 + 阻力训练 + 水中有氧运动疗法 + 太极 + 气功 + 瑜伽 + 普拉提 + 跑 + 跳 + 技能游戏 + 高强度间歇训练 + 呼吸训练 + 平衡训练 + 柔韧训练 + 功能性运动 + 稳定性训练 + 功能体操）AND SU=（痴呆 + 神经退行性病）AND SU=（抑郁）

VIP

主题词：（运动 + 拉伸 + 伸展运动 + 水疗 + 阻力训练 + 水中有氧运动疗法 + 太极 + 气功 + 瑜伽 + 普拉提 + 跑 + 跳 + 技能游戏 + 高强度间歇训练 + 呼吸训练 + 平衡训练 + 柔韧训练 + 功能性运动 + 稳定性训练 + 功能体操）AND主题词：（痴呆 + 神经退行性病）AND主题词：（抑郁）

Wanfang

主题:(运动 OR 拉伸 OR 伸展运动 OR 水疗 OR 阻力训练 OR 水中有氧运动疗法 OR 太极 OR 气功 OR 瑜伽 OR 普拉提 OR 跑 OR跳 OR 技能游戏 OR 高强度间歇训练 OR 呼吸训练 OR 平衡训练 OR 柔韧训练 OR 功能性运动 OR 稳定性训练 OR 功能体操) And 主题:(痴呆 OR 神经退行性病) And 主题:(抑郁)

SinoMed

**#1** "痴呆"[不加权:扩展] + "痴呆"[常用字段:智能] + "神经退行性病"[常用字段:智能]

**#2** "抑郁"[不加权:扩展] + "抑郁"[常用字段:智能]

**#3** "运动(Motion)"[不加权:扩展] + "运动"[常用字段:智能] + "拉伸"[常用字段:智能] + "拉伸运动"[常用字段:智能] + "水疗"[常用字段:智能] + "阻力训练"[常用字段:智能] + "水中有氧运动疗法"[常用字段:智能] + "太极"[常用字段:智能] + "气功"[常用字段:智能] + "瑜伽"[常用字段:智能] + "普拉提"[常用字段:智能] + "跑"[常用字段:智能] + "跳"[常用字段:智能] + "技能游戏"[常用字段:智能] + "高强度间歇训练"[常用字段:智能] + "呼吸训练"[常用字段:智能] + "平衡训练"[常用字段:智能] + "柔韧训练"[常用字段:智能] + "功能性训练"[常用字段:智能]

**#4** #1 AND #2 AND #3

Pubmed

**#1** (((((((((((((dementia[Title/Abstract]) OR (Dementias[Title/Abstract])) OR (Amentia[Title/Abstract])) OR (Amentias[Title/Abstract])) OR (Senile Paranoid Dementia[Title/Abstract])) OR (Dementias, Senile Paranoid[Title/Abstract])) OR (Paranoid Dementia, Senile[Title/Abstract])) OR (Paranoid Dementias, Senile[Title/Abstract])) OR (Senile Paranoid Dementias[Title/Abstract])) OR (Familial Dementia[Title/Abstract])) OR (Dementia, Familial[Title/Abstract])) OR (Dementias, Familial[Title/Abstract])) OR (Familial Dementias[Title/Abstract])) OR ("Dementia"[Mesh])

**#2** ((((((((((((((((Neurodegenerative disease[Title/Abstract]) OR (Neurodegenerative disease[Title/Abstract])) OR (Degenerative Neurologic Disorder[Title/Abstract])) OR (Neurologic Disorder, Degenerative[Title/Abstract])) OR (Neurologic Disorders, Degenerative[Title/Abstract])) OR (Nervous System Degenerative Diseases[Title/Abstract])) OR (Neurodegenerative Disorders[Title/Abstract])) OR (Neurodegenerative Disorder[Title/Abstract])) OR (Degenerative Diseases, Nervous System[Title/Abstract])) OR (Degenerative Diseases, Neurologic[Title/Abstract])) OR (Neurologic Degenerative Disease[Title/Abstract])) OR (Neurologic Degenerative Conditions[Title/Abstract])) OR (Degenerative Condition, Neurologic[Title/Abstract])) OR (Degenerative Conditions, Neurologic[Title/Abstract])) OR (Neurologic Degenerative Condition[Title/Abstract])) OR (Neurologic Degenerative Diseases[Title/Abstract])) OR ("Neurodegenerative Diseases"[Mesh])

**#3** #1 OR #2

**#4** ((((((depression[Title/Abstract]) OR (Depressive Symptoms[Title/Abstract])) OR (Depressive Symptom[Title/Abstract])) OR (Symptom, Depressive[Title/Abstract])) OR (Emotional Depression[Title/Abstract])) OR (Depression, Emotional[Title/Abstract])) OR ("Depression"[Mesh])

**#5** (((((((((((((((((((((((Exercise Therapy[Title/Abstract]) OR (Rehabilitation Exercise[Title/Abstract])) OR (Exercise, Rehabilitation[Title/Abstract])) OR (Exercises, Rehabilitation[Title/Abstract])) OR (Rehabilitation Exercises[Title/Abstract])) OR (Therapy, Exercise[Title/Abstract])) OR (Exercise Therapies[Title/Abstract])) OR (Therapies, Exercise[Title/Abstract])) OR (Remedial Exercise[Title/Abstract])) OR (Exercise, Remedial[Title/Abstract])) OR (Exercises, Remedial[Title/Abstract])) OR (Remedial Exercises[Title/Abstract])) OR ("Exercise Therapy"[Mesh])) OR (((((((((((((((((((((((((((((((((((Muscle Stretching Exercises[Title/Abstract]) OR (Exercise, Muscle Stretching[Title/Abstract])) OR (Muscle Stretching Exercise[Title/Abstract])) OR (Active Stretching[Title/Abstract])) OR (Stretching, Active[Title/Abstract])) OR (Static-Active Stretching[Title/Abstract])) OR (Static Active Stretching[Title/Abstract])) OR (Stretching, Static-Active[Title/Abstract])) OR (Proprioceptive Neuromuscular Facilitation (PNF) Stretching[Title/Abstract])) OR (Proprioceptive Neuromuscular Facilitation[Title/Abstract])) OR (Neuromuscular Facilitation, Proprioceptive[Title/Abstract])) OR (Proprioceptive Neuromuscular Facilitations[Title/Abstract])) OR (PNF Stretching[Title/Abstract])) OR (PNF Stretchings[Title/Abstract])) OR (Stretching, PNF[Title/Abstract])) OR (PNF Stretching Exercise[Title/Abstract])) OR (Exercise, PNF Stretching[Title/Abstract])) OR (PNF Stretching Exercises[Title/Abstract])) OR (Stretching Exercise, PNF[Title/Abstract])) OR (Passive Stretching[Title/Abstract])) OR (Stretching, Passive[Title/Abstract])) OR (Static-Passive Stretching[Title/Abstract])) OR (Static Passive Stretching[Title/Abstract])) OR (Stretching, Static-Passive[Title/Abstract])) OR (Relaxed Stretching[Title/Abstract])) OR (Stretching, Relaxed[Title/Abstract])) OR (Static Stretching[Title/Abstract])) OR (Stretching, Static[Title/Abstract])) OR (Isometric Stretching[Title/Abstract])) OR (Stretching, Isometric[Title/Abstract])) OR (Dynamic Stretching[Title/Abstract])) OR (Stretching, Dynamic[Title/Abstract])) OR (Ballistic Stretching[Title/Abstract])) OR (Stretching, Ballistic[Title/Abstract])) OR ("Muscle Stretching Exercises"[Mesh]))) OR (((((((((((((((Aquatic Therapy[Title/Abstract]) OR (Therapy, Aquatic[Title/Abstract])) OR (Pool Therapy[Title/Abstract])) OR (Therapy, Pool[Title/Abstract])) OR (Ai Chi Therapy[Title/Abstract])) OR (Therapies, Ai Chi[Title/Abstract])) OR (Therapy, Ai Chi[Title/Abstract])) OR (Water Tai Chi Therapy[Title/Abstract])) OR (Aquatic Exercise Therapy[Title/Abstract])) OR (Exercise Therapy, Aquatic[Title/Abstract])) OR (Therapy, Aquatic Exercise[Title/Abstract])) OR (Water Exercise Therapy[Title/Abstract])) OR (Exercise Therapy, Water[Title/Abstract])) OR (Therapy, Water Exercise[Title/Abstract])) OR ("Aquatic Therapy"[Mesh]))) OR (((((((((((((((((((((((((Resistance Training[Title/Abstract]) OR (Training, Resistance[Title/Abstract])) OR (Strength Training[Title/Abstract])) OR (Training, Strength[Title/Abstract])) OR (Weight-Lifting Strengthening Program[Title/Abstract])) OR (Strengthening Programs, Weight-Lifting[Title/Abstract])) OR (Strengthening Program, Weight-Lifting[Title/Abstract])) OR (Weight Lifting Strengthening Program[Title/Abstract])) OR (Weight-Lifting Strengthening Programs[Title/Abstract])) OR (Weight-Lifting Exercise Program[Title/Abstract])) OR (Exercise Programs, Weight-Lifting[Title/Abstract])) OR (Exercise Program, Weight-Lifting[Title/Abstract])) OR (Weight Lifting Exercise Program[Title/Abstract])) OR (Weight-Lifting Exercise Programs[Title/Abstract])) OR (Weight-Bearing Strengthening Program[Title/Abstract])) OR (Strengthening Programs, Weight-Bearing[Title/Abstract])) OR (Strengthening Program, Weight-Bearing[Title/Abstract])) OR (Weight Bearing Strengthening Program[Title/Abstract])) OR (Weight-Bearing Strengthening Programs[Title/Abstract])) OR (Weight-Bearing Exercise Program[Title/Abstract])) OR (Exercise Programs, Weight-Bearing[Title/Abstract])) OR (Exercise Program, Weight-Bearing[Title/Abstract])) OR (Weight Bearing Exercise Program[Title/Abstract])) OR (Weight-Bearing Exercise Programs[Title/Abstract])) OR ("Resistance Training"[Mesh]))) OR ((Yoga[Title/Abstract]) OR ("Yoga"[Mesh]))) OR ((((((((((((Tai Ji[Title/Abstract]) OR (Tai-ji[Title/Abstract])) OR (Tai Chi[Title/Abstract])) OR (Chi, Tai[Title/Abstract])) OR (Tai Chi Chuan[Title/Abstract])) OR (Taiji[Title/Abstract])) OR (Taijiquan[Title/Abstract])) OR (T'ai Chi[Title/Abstract])) OR (Tai Ji Quan[Title/Abstract])) OR (Ji Quan, Tai[Title/Abstract])) OR (Quan, Tai Ji[Title/Abstract])) OR ("Tai Ji"[Mesh]))) OR (((((((((Exercise Movement Techniques[Title/Abstract]) OR (Movement Techniques, Exercise[Title/Abstract])) OR (Exercise Movement Technics[Title/Abstract])) OR (Pilates-Based Exercises[Title/Abstract])) OR (Exercises, Pilates-Based[Title/Abstract])) OR (Pilates Based Exercises[Title/Abstract])) OR (Pilates Training[Title/Abstract])) OR (Training, Pilates[Title/Abstract])) OR ("Exercise Movement Techniques"[Mesh]))) OR (((Running[Title/Abstract]) OR (Runnings[Title/Abstract])) OR ("Running"[Mesh]))) OR (((jogging[Title/Abstract]) OR (joggings[Title/Abstract])) OR ("Jogging"[Mesh]))) OR ((((((Breathing Exercises[Title/Abstract]) OR (Exercise, Breathing[Title/Abstract])) OR (Respiratory Muscle Training[Title/Abstract])) OR (Muscle Training, Respiratory[Title/Abstract])) OR (Training, Respiratory Muscle[Title/Abstract])) OR ("Breathing Exercises"[Mesh]))) OR ((((((((((((((High-Intensity Interval Training[Title/Abstract]) OR (High Intensity Interval Training[Title/Abstract])) OR (High-Intensity Interval Trainings[Title/Abstract])) OR (Interval Training, High-Intensity[Title/Abstract])) OR (Interval Trainings, High-Intensity[Title/Abstract])) OR (Training, High-Intensity Interval[Title/Abstract])) OR (Trainings, High-Intensity Interval[Title/Abstract])) OR (High-Intensity Intermittent Exercise[Title/Abstract])) OR (Exercise, High-Intensity Intermittent[Title/Abstract])) OR (Exercises, High-Intensity Intermittent[Title/Abstract])) OR (High-Intensity Intermittent Exercises[Title/Abstract])) OR (Sprint Interval Training[Title/Abstract])) OR (Sprint Interval Trainings[Title/Abstract])) OR ("High-Intensity Interval Training"[Mesh]))) OR ((((((((Circuit-Based Exercise[Title/Abstract]) OR (Circuit Based Exercise[Title/Abstract])) OR (Circuit-Based Exercises[Title/Abstract])) OR (Exercise, Circuit-Based[Title/Abstract])) OR (Exercises, Circuit-Based[Title/Abstract])) OR (Circuit Training[Title/Abstract])) OR (Training, Circuit[Title/Abstract])) OR ("Circuit-Based Exercise"[Mesh]))

**#6** #3 AND #4 AND #5

Cochrane

**#1** MeSH descriptor: [Dementia] explode all trees

**#2** Dementia OR Amentias OR Dementias OR Amentia OR Dementia, Familial OR Familial Dementias OR Familial Dementia OR Dementias, Familial OR Senile Paranoid Dementias OR Dementias, Senile Paranoid OR Senile Paranoid Dementia OR Paranoid Dementia, Senile OR Paranoid Dementias, Senile

**#3** #1 OR #2

**#4** MeSH descriptor: [Neurodegenerative Diseases] explode all trees

**#5** Neurodegenerative disease OR Degenerative Diseases, Spinal Cord OR Degenerative Diseases, Central Nervous System OR Degenerative Condition, Neurologic OR Degenerative Conditions, Neurologic OR Neurologic Degenerative Condition OR Neurologic Diseases, Degenerative OR Neurologic Degenerative Conditions OR Neurodegenerative Disorder OR Neurodegenerative Disease OR Degenerative Neurologic Disorders OR Neurologic Degenerative Diseases OR Neurodegenerative Disorders OR Degenerative Diseases, Nervous System OR Degenerative Diseases, Neurologic OR Neurologic Disorders, Degenerative OR Degenerative Neurologic Disorder OR Neurologic Disease, Degenerative OR Nervous System Degenerative Diseases OR Neurologic Degenerative Disease OR Degenerative Neurologic Diseases OR Degenerative Neurologic Disease OR Neurologic Disorder, Degenerative

**#6** #4 OR #5

**#7** #3 OR #6

**#8** MeSH descriptor: [Depression] explode all trees

**#9** Depression OR Emotional Depression OR Depressive Symptoms OR Depressive Symptom OR Symptom, Depressive OR Depression, Emotional

**#10** #8 or #9

**#11** MeSH descriptor: [Exercise Therapy] explode all trees

**#12** Exercise Therapy OR Remedial Exercises OR Rehabilitation Exercises OR Exercises, Remedial OR Therapy, Exercise OR Exercise, Rehabilitation OR Rehabilitation Exercise OR Therapies, Exercise OR Exercise Therapies OR Remedial Exercise OR Exercises, Rehabilitation OR Exercise, Remedial

**#13** #11 OR #12

**#14** MeSH descriptor: [Muscle Stretching Exercises] explode all trees

**#15** Muscle Stretching Exercise OR Static Stretching OR Stretching, Static OR Ballistic Stretching OR Stretching, Ballistic OR Stretching, Passive OR Stretching, Relaxed OR Passive Stretching OR Stretching, Static-Passive OR Relaxed Stretching OR Static-Passive Stretching OR Static Passive Stretching OR Proprioceptive Neuromuscular Facilitations OR PNF Stretching Exercise OR PNF Stretching Exercises OR Stretching Exercise, PNF OR Neuromuscular Facilitation, Proprioceptive OR Proprioceptive Neuromuscular Facilitation OR Exercise, PNF Stretching OR PNF Stretching OR Proprioceptive Neuromuscular Facilitation (PNF) Stretching OR PNF Stretchings OR Stretching, PNF OR Dynamic Stretching OR Stretching, Dynamic OR Muscle Stretching Exercise OR Exercise, Muscle Stretching OR Stretching, Isometric OR Isometric Stretching OR Static Active Stretching OR Active Stretching OR Stretching, Active OR Static-Active Stretching OR Stretching, Static-Active

**#16** #14 OR #15

**#17** MeSH descriptor: [Aquatic Therapy] explode all trees

**#18** Aquatic Therapy OR Aquatic Exercise Therapy OR Water Exercise Therapy OR Therapy, Water Exercise OR Therapy, Aquatic Exercise OR Exercise Therapy, Aquatic OR Exercise Therapy, Water OR Therapy, Pool OR Pool Therapy OR Therapies, Ai Chi OR Therapy, Ai Chi OR Water Tai Chi Therapy OR Ai Chi Therapy OR Therapy, Aquatic

**#19** #17 OR #18

**#20** MeSH descriptor: [Resistance Training] explode all trees

**#21** Resistance Training OR Training, Resistance OR Strength Training OR Training, Strength OR Weight-Bearing Exercise Program OR Weight Bearing Exercise Program OR Weight-Bearing Strengthening Program OR Weight-Bearing Exercise Programs OR Exercise Program, Weight-Bearing OR Strengthening Program, Weight-Bearing OR Strengthening Programs, Weight-Bearing OR Weight Bearing Strengthening Program OR Exercise Programs, Weight-Bearing OR Weight-Bearing Strengthening Programs OR Weight-Lifting Exercise Programs OR Weight Lifting Exercise Program OR Exercise Program, Weight-Lifting OR Weight-Lifting Exercise Program OR Weight Lifting Strengthening Program OR Weight-Lifting Strengthening Program OR Strengthening Programs, Weight-Lifting OR Weight-Lifting Strengthening Programs OR Exercise Programs, Weight-Lifting OR Strengthening Program, Weight-Lifting

**#22** #20 OR #21

**#23** MeSH descriptor: [Yoga] explode all trees

**#24** Yoga

**#25** #23 OR #24

**#26** MeSH descriptor: [Tai Ji] explode all trees

**#27** Tai Ji OR  Tai Chi Chuan OR Tai-ji OR Taijiquan OR T'ai Chi OR Quan, Tai Ji OR Tai Ji Quan OR Chi, Tai OR Tai Chi OR Ji Quan, Tai OR Taiji

**#28** #26 OR #27

**#29** MeSH descriptor: [Exercise Movement Techniques] explode all trees

**#30** Exercise Movement Techniques OR Exercise Movement Technics OR Movement Techniques, Exercise OR Pilates Based Exercises OR Exercises, Pilates-Based OR Pilates Training OR Pilates-Based Exercises OR Training, Pilates

**#31** #29 OR #30

**#32** MeSH descriptor: [Running] explode all trees

**#33** Running OR Runnings

**#34** #32 OR #33

**#35** MeSH descriptor: [Jogging] explode all trees

**#36** Jogging OR Joggings

**#37** #35 OR #36

**#38** MeSH descriptor: [Breathing Exercises] explode all trees

**#39** Breathing Exercises OR Exercise, Breathing OR Respiratory Muscle Training OR Training, Respiratory Muscle OR Muscle Training, Respiratory

**#40** #38 OR #39

**#41** MeSH descriptor: [High-Intensity Interval Training] explode all trees

**#42** High-Intensity Interval Training OR  Exercise, High-Intensity Intermittent OR High-Intensity Interval Trainings OR High-Intensity Intermittent Exercises OR Training, High-Intensity Interval OR Trainings, High-Intensity Interval OR Interval Training, High-Intensity OR High Intensity Interval Training OR Interval Trainings, High-Intensity OR High-Intensity Intermittent Exercise OR Exercises, High-Intensity Intermittent OR Sprint Interval Trainings OR Sprint Interval Training

**#43** #41 OR #42

**#44** MeSH descriptor: [Circuit-Based Exercise] explode all trees

**#45** Circuit-Based Exercise OR Training, Circuit OR Circuit Training OR Circuit Based Exercise OR Circuit-Based Exercises OR Exercises, Circuit-Based OR Exercise, Circuit-Based

**#46** #44 OR #45

**#47** #13 OR #16 OR #19 OR #22 OR #25 OR #28 OR #31 OR #34 OR #37 OR #40 OR #43 OR #46

**#48** #7 AND #10 AND #47

Embase

(('amentia'ti,ab OR 'demention'ti,ab OR 'dementia'ti,ab) OR ('neurodegenerative disease'ti,ab OR 'neurodegenerative diseases'ti,ab OR 'degenerative disease'ti,ab)) AND ('central depression'ti,ab OR 'clinical depression'ti,ab OR 'depressive disease'ti,ab OR 'depressive disorder'ti,ab OR 'depressive episode'ti,ab OR 'depressive illness'ti,ab OR 'depressive personality disorder'ti,ab OR 'depressive state'ti,ab OR 'depressive symptom'ti,ab OR 'depressive syndrome'ti,ab OR 'depressivity'ti,ab OR 'mental depression'ti,ab OR 'parental depression'ti,ab OR 'depression'ti,ab) AND ('corrective exercise'ti,ab OR 'exercise movement techniques'ti,ab OR 'exercise therapy'ti,ab OR 'exercise treatment'ti,ab OR 'kinesiotherapeutic intervention'ti,ab OR 'kinesiotherapeutic method'ti,ab OR 'kinesiotherapeutic procedure'ti,ab OR 'kinesiotherapeutic technique'ti,ab OR 'kinesiotherapeutical treatment'ti,ab OR 'kinesitherapeutic exercises'ti,ab OR 'kinesitherapeutic intervention'ti,ab OR 'kinesitherapeutic method'ti,ab OR 'kinesitherapeutic methodology'ti,ab OR 'kinesitherapeutic procedure'ti,ab OR 'kinesitherapeutic technique'ti,ab OR 'kinesitherapeutic treatment'ti,ab OR 'kinesitherapeutical treatment'ti,ab OR 'kinesitherapy'ti,ab OR 'SKTM (specialized kinesitherapeutic methodology)'ti,ab OR 'specialised kinesitherapeutic methodology'ti,ab OR 'specialized kinesitherapeutic methodology'ti,ab OR 'therapeutic exercise'ti,ab OR 'therapy, exercise'ti,ab OR 'treatment, exercise'ti,ab OR 'kinesiotherapy'ti,ab) OR (‘Stretching’ti,ab) OR ('aquatic exercise therapy'ti,ab OR 'pool exercise therapy'ti,ab OR 'pool therapy'ti,ab OR 'water exercise therapy'ti,ab OR 'aquatic therapy'ti,ab) OR ('resistance exercise'ti,ab OR 'resistance exercise training'ti,ab OR 'resistance-type exercise'ti,ab OR 'resistance-type training'ti,ab OR 'strength training'ti,ab OR 'strength-type exercise'ti,ab OR 'strength-type training'ti,ab OR 'resistance training') OR ('yogic meditation'ti,ab OR 'yoga') OR ('Tai Chi Chuan'ti,ab OR 'Tai Ji'ti,ab OR 'Taiji quan'ti,ab OR 'Taijiquan'ti,ab OR 'Tai Chi'ti,ab) OR ('pilates exercise'ti,ab OR 'pilates'ti,ab) OR (‘Running’ti,ab) OR (‘Jogging’ti,ab) OR ('breathing exercises'ti,ab OR 'breathing therapy'ti,ab OR 'chest physical therapy'ti,ab OR 'chest physiotherapy'ti,ab or 'exercise, breathing'ti,ab OR 'exercise, respiratory'ti,ab OR 'respiration exercise'ti,ab OR 'respiration therapy'ti,ab OR 'respiratory exercise'yi,ab OR 'respiratory physiotherapy'ti,ab OR 'breathing exercise'ti,ab) OR ('breathing exercises'):ab,ti OR (('breathing therapy'):ab,ti) OR (('chest physical therapy'):ab,ti) OR (('chest physiotherapy'):ab,ti) OR (('exercise, breathing'):ab,ti) OR (('exercise, respiratory'):ab,ti) OR (('respiration exercise'):ab,ti) OR (('respiration therapy'):ab,ti) OR (('respiratory exercise'):ab,ti) OR (('respiratory physiotherapy'):ab,ti) OR (('breathing exercise'):ab,ti) OR ('high-intensity intermittent exercise'ti,ab OR 'high-intensity intermittent training'ti,ab OR 'high-intensity interval exercise'ti,ab OR 'high-intensity interval training'ti,ab OR 'HIIE (exercise)'ti,ab OR 'HIIT'ti,ab OR 'intermittent high-intensity training'ti,ab OR 'interval high-intensity training'ti,ab OR 'high intensity interval training'ti,ab) OR ('circuit-based exercise'ti,ab OR 'circuit-based training'ti,ab OR 'circuit-type exercise'ti,ab OR 'circuit-type training'ti,ab OR 'circuit training'ti,ab)

Web of Science

((TS=(Dementia OR Dementias OR Amentia OR Amentias OR Senile Paranoid Dementia OR Dementias, Senile Paranoid OR Paranoid Dementia, Senile OR Paranoid Dementias, Senile OR Senile Paranoid Dementias OR Familial Dementia OR Dementia, Familial OR Dementias, Familial OR Familial Dementias)) OR (TS=(Neurodegenerative disease OR Neurodegenerative disease OR Degenerative Neurologic Disorder OR Neurologic Disorder, Degenerative OR Neurologic Disorders, Degenerative OR Nervous System Degenerative Diseases OR Neurodegenerative Disorders OR Neurodegenerative Disorder OR Degenerative Diseases, Nervous System OR Degenerative Diseases, Neurologic OR Neurologic Degenerative Disease OR Neurologic Degenerative Conditions OR Degenerative Condition, Neurologic OR Degenerative Conditions, Neurologic OR Neurologic Degenerative Condition OR Neurologic Degenerative Diseases))) AND (TS=(Depression OR Depressive Symptoms OR Depressive Symptom OR Symptom, Depressive OR Emotional Depression OR Depression, Emotional) ) AND TS=(Exercise Therapy OR Rehabilitation Exercise OR Exercise, Rehabilitation OR Exercises, Rehabilitation OR Rehabilitation Exercises OR Therapy, Exercise OR Exercise Therapies OR Therapies, Exercise OR Remedial Exercise OR Exercise, Remedial OR Exercises, Remedial OR Remedial Exercises) OR TS=(Muscle Stretching Exercises OR Exercise, Muscle Stretching OR Muscle Stretching Exercise OR Active Stretching OR Stretching, Active OR Static-Active Stretching OR Static Active Stretching OR Stretching, Static-Active OR Proprioceptive Neuromuscular Facilitation (PNF) Stretching OR Proprioceptive Neuromuscular Facilitation OR Neuromuscular Facilitation, Proprioceptive OR Proprioceptive Neuromuscular Facilitations OR PNF Stretching OR PNF Stretchings OR Stretching, PNF OR PNF Stretching Exercise OR Exercise, PNF Stretching OR PNF Stretching Exercises OR Stretching Exercise, PNF OR Passive Stretching OR Stretching, Passive OR Static-Passive Stretching OR Static Passive Stretching OR Stretching, Static-Passive OR Relaxed Stretching OR Stretching, Relaxed OR Static Stretching OR Stretching, Static OR Isometric Stretching OR Stretching, Isometric OR Dynamic Stretching OR Stretching, Dynamic OR Ballistic Stretching OR Stretching, Ballistic) OR TS=(Aquatic Therapy OR Therapy, Aquatic OR Pool Therapy OR Therapy, Pool OR Ai Chi Therapy OR Therapies, Ai Chi OR Therapy, Ai Chi OR Water Tai Chi Therapy OR Aquatic Exercise Therapy OR Exercise Therapy, Aquatic OR Therapy, Aquatic Exercise OR Water Exercise Therapy OR Exercise Therapy, Water OR Therapy, Water Exercise) OR TS=(Resistance Training OR Training, Resistance OR Strength Training OR Training, Strength OR Weight-Lifting Strengthening Program OR Strengthening Programs, Weight-Lifting OR Strengthening Program, Weight-Lifting OR Weight Lifting Strengthening Program OR Weight-Lifting Strengthening Programs OR Weight-Lifting Exercise Program OR Exercise Programs, Weight-Lifting OR Exercise Program, Weight-Lifting OR Weight Lifting Exercise Program OR Weight-Lifting Exercise Programs OR Weight-Bearing Strengthening Program OR Strengthening Programs, Weight-Bearing OR Strengthening Program, Weight-Bearing OR Weight Bearing Strengthening Program OR Weight-Bearing Strengthening Programs OR Weight-Bearing Exercise Program OR Exercise Programs, Weight-Bearing OR Exercise Program, Weight-Bearing OR Weight Bearing Exercise Program OR Weight-Bearing Exercise Programs) OR TS=(Yoga) OR TS=(Tai Ji OR Tai-ji OR Tai Chi OR Chi, Tai OR Tai Chi Chuan OR Taiji OR Taijiquan OR T'ai Chi OR Tai Ji Quan OR Ji Quan, Tai OR Quan, Tai Ji) OR TS=(Exercise Movement Techniques OR Movement Techniques, Exercise OR Exercise Movement Technics OR Pilates-Based Exercises OR Exercises, Pilates-Based OR Pilates Based Exercises OR Pilates Training OR Training, Pilates) OR TS=(Running OR Runnings) OR TS=(Jogging OR Joggings) OR TS=(Breathing Exercises OR Exercise, Breathing OR Respirat+y Muscle Training OR Muscle Training, Respirat+y OR Training, Respirat+y Muscle) OR TS=(High-Intensity Interval Training OR High Intensity Interval Training OR High-Intensity Interval Trainings OR Interval Training, High-Intensity OR Interval Trainings, High-Intensity OR Training, High-Intensity Interval OR Trainings, High-Intensity Interval OR High-Intensity Intermittent Exercise OR Exercise, High-Intensity Intermittent OR Exercises, High-Intensity Intermittent OR High-Intensity Intermittent Exercises OR Sprint Interval Training + Sprint Interval Trainings) OR TS=(Circuit-Based Exercise OR Circuit Based Exercise OR Circuit-Based Exercises OR Exercise, Circuit-Based OR Exercises, Circuit-Based OR Circuit Training OR Training, Circuit))
